# Measuring cell-to-cell expression variability in single-cell RNA-sequencing data: a comparative analysis and applications to B cell aging

**DOI:** 10.1186/s13059-023-03036-2

**Published:** 2023-10-20

**Authors:** Huiwen Zheng, Jan Vijg, Atefeh Taherian Fard, Jessica Cara Mar

**Affiliations:** 1 Australian Institute for Bioengineering and Nanotechnology, The University of Queensland, Brisbane, QLD Australia; 2 Department of Genetics, Albert Einstein College of Medicine, Bronx, NY 10461 USA; 3 Center for Single-Cell Omics, School of Public Health, Shanghai Jiao Tong University School of Medicine, Shanghai, 200025 China

**Keywords:** Cell-to-cell variability, B lymphocytes differentiation, Aging, Single-cell RNA-seq, Evaluation framework

## Abstract

**Background:**

Single-cell RNA-sequencing (scRNA-seq) technologies enable the capture of gene expression heterogeneity and consequently facilitate the study of cell-to-cell variability at the cell type level. Although different methods have been proposed to quantify cell-to-cell variability, it is unclear what the optimal statistical approach is, especially in light of challenging data structures that are unique to scRNA-seq data like zero inflation.

**Results:**

We systematically evaluate the performance of 14 different variability metrics that are commonly applied to transcriptomic data for measuring cell-to-cell variability. Leveraging simulations and real datasets, we benchmark the metric performance based on data-specific features, sparsity and sequencing platform, biological properties, and the ability to recapitulate true levels of biological variability based on known gene sets. Next, we use scran, the metric with the strongest all-round performance, to investigate changes in cell-to-cell variability that occur during B cell differentiation and the aging processes. The analysis of primary cell types from hematopoietic stem cells (HSCs) and B lymphopoiesis reveals unique gene signatures with consistent patterns of variable and stable expression profiles during B cell differentiation which highlights the significance of these methods. Identifying differentially variable genes between young and old cells elucidates the regulatory changes that may be overlooked by solely focusing on mean expression changes and we investigate this in the context of regulatory networks.

**Conclusions:**

We highlight the importance of capturing cell-to-cell gene expression variability in a complex biological process like differentiation and aging and emphasize the value of these findings at the level of individual cell types.

**Supplementary Information:**

The online version contains supplementary material available at 10.1186/s13059-023-03036-2.

## Background

Cells are the basic units of life, and heterogeneity in gene expression is something that exists between cells, even in populations of genetically identical cells. At the molecular level, cell-to-cell variability results from cells that cancel out intrinsic noise while amplifying regulated variability [1]. The demonstrated link between cell-to-cell variability and the propagation of gene expression through regulatory networks makes it critically important to model changes in cell-to-cell variability as a means to identify regulators that may have been overlooked by focusing on changes in average expression only.

Advances in single-cell RNA sequencing (scRNA-seq) technologies have pushed the boundaries of the resolution at which gene expression measurements can now be obtained from single cells [2]. The popularity of scRNA-seq datasets has renewed interest in gene expression variability and what this metric can reveal about underlying regulatory processes [3, 4]. Although cell-to-cell variability is a straightforward concept, a range of terms has been used to describe it, including cellular heterogeneity, transcriptional variability, gene expression variability, or transcriptional noise. There has been an even greater number of metrics used to measure cell-to-cell variability that follow their own distinct statistical approaches. This is seemingly problematic because each study of cell-to-cell variability adopts its own specific quantitative approach for the analysis. Without an understanding of which variability metrics have the strongest performance for scRNA-seq data, it is possible that these studies are using variability estimates in a sub-optimal way, therefore making it difficult to model cell-to-cell variability in complex biological processes such as aging.

Measuring gene expression variability is challenging for scRNA-seq data because of the typical characteristics that this sequencing approach generates that create additional limitations for modeling transcript counts and the true levels of cell-to-cell variability. For example, sparsity, which is in part driven by stochastic gene expression, makes it challenging to estimate cell-to-cell variability because many traditional summary statistics cannot handle the increased frequency of zeros. Additionally, low mRNA capture efficiency and low sequencing depth contribute to sparsity that can make it challenging to capture the sufficient and equal cell type sizes that are required to model true gene expression variability. On the other hand, genes with very low read counts tend to have more variable expression, resulting in a strong mean–variance relationship [5]. Therefore, if this relationship is not being accounted for, changes in the measured variability may be driven by fluctuations of lowly expressed genes rather than the true variability. While we understand these issues in the context of how we model scRNA-seq data through typical workflows, modeling variability is in some ways more complicated because the variability is influenced to a greater degree by aspects like sample size, than statistics based on average expression [6].

Studying aging showcases the value of what can be learned from gene expression variability of scRNA-seq data because aging is a genetically regulated program that is executed by a series of cellular and molecular factors and influenced by stochastic processes. For example, bone marrow has been intensively investigated as it is the primary site to produce hematopoietic stem cells (HSCs) which give rise to all the cells involved in the immune response. Throughout life, the function of the bone marrow adapts to match the fluctuating demands of the organism and impacts HSCs’ self-renewing [7]. The long-term reconstituting HSCs can lead to phenotypically and transcriptionally distinct short-term HSCs that lack durable self-renewal, which can progressively generate lineage-restricted progenitors and mature cells of the myeloid, lymphoid, and megaerythroid lineages [8]. Furthermore, the reduction of the regenerative capacity of the stem cells and B lymphoid lineages generation rates during aging results in narrowed clonotypic diversity [9] which presumably must impact cell-to-cell variability of gene expression. These observations highlight the importance of profiling cell-to-cell variability to characterize the developmental process of aging HSCs and B lymphoid lineages.

Age-dependent changes in gene expression variability are difficult to address because of inherent noise and experimental factors that may influence variability [10]. Large databases like Tabula-Muris-Senis (TMS) [11] and GTEx [12] provide the opportunity to expand our knowledge of how cell-to-cell variability changes during aging in various organs and tissues. Several studies have reported that gene expression variability increases during aging such as mouse heart cardiomyocytes [13] and human pancreatic cells [14]. However, these increases in variability during aging are not consistently observed across all tissue or cell types, e.g., in aging hematopoietic cell types and mouse brains [15, 16]. These inconsistencies may be due to genuine biological effects, differences in the experimental factors inherent to the design, or the choice of the analysis approach of these datasets. Resolving these inconsistencies to identify what patterns of gene expression variability can be generalized between conditions to help provide insight into complex processes like aging.

In this study, we conduct a systematic evaluation of a wide range of metrics that are used for measuring gene expression variability in transcriptomic data. The focus of the evaluation was to identify which variability metric had the strongest performance when evaluated against a set of different biological and data-specific features that are relevant to scRNA-seq data. The winner of this benchmarking exercise, scran, was used to investigate the role of cell-to-cell variability in identifying regulatory relationships underlying the aging process on HSCs and B lymphoid lineages sourced from the TMS database. We observed cellular heterogeneity changes that were cell-type-specific for B lymphoid differentiation during aging. These results aligned with existing underlying biology and provided additional evidence for stem cell exhaustion and a decline in B lymphomagenesis in aging. Furthermore, we also developed a new method to identify differentially variable genes in aged HSCs and B cells, which showed distinct regulatory networks with a shared transcription factor. Taken together, our results provide ample evidence that studying cell-to-cell variability at the cell type level has important implications for understanding aging cells that undergo cell-type-specific differentiation processes.

## Results

### Overview of the metric comparison framework

**Fig. 1:**
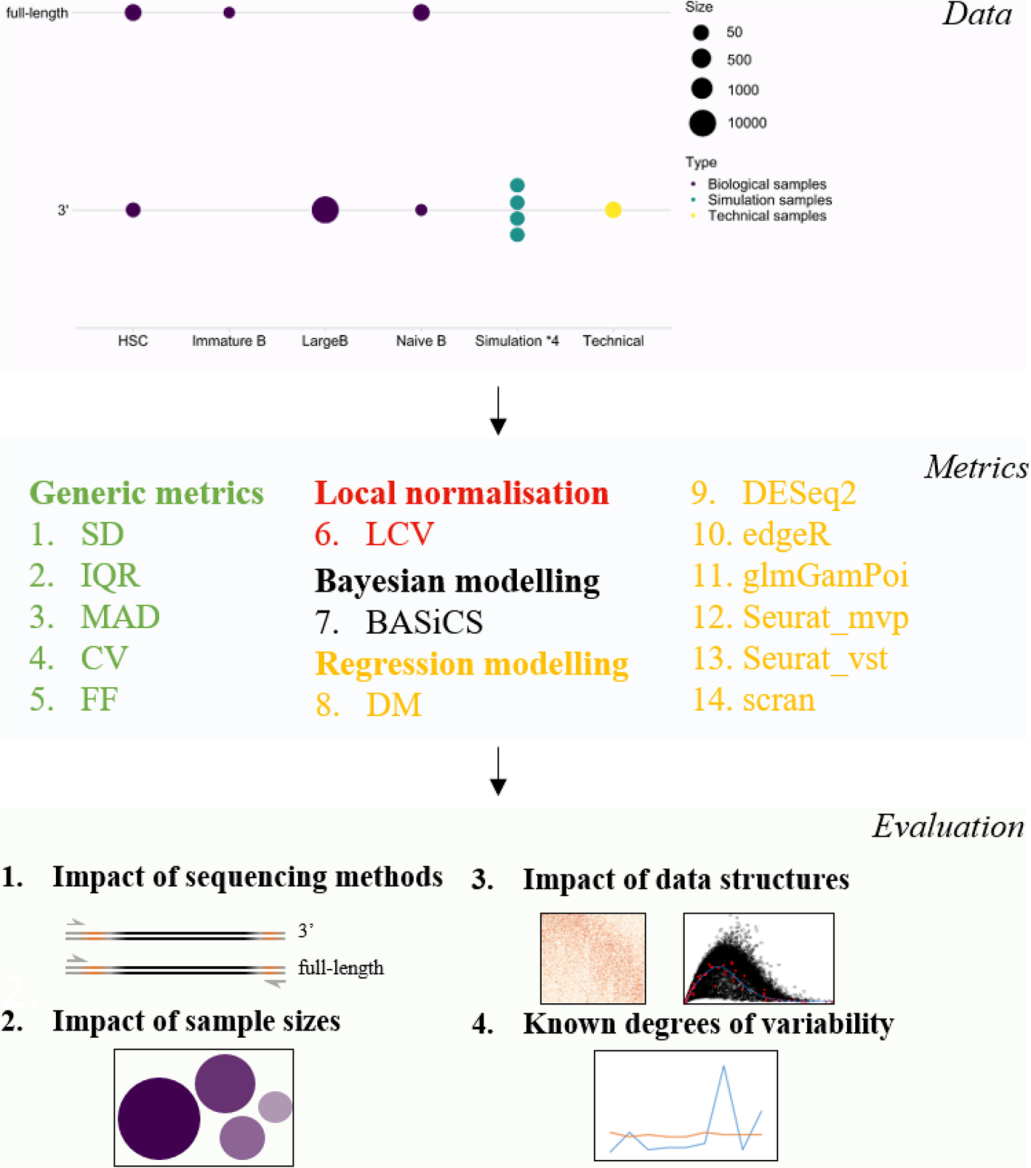
Schematic summaries of the benchmark workflow to evaluate the performance of cell-to-cell variability metrics for scRNA-seq data. A panel of 14 metrics that perform the estimation of cell-to-cell variability were evaluated on both experimentally-derived and simulated data from two main sequencing platforms with different sample sizes. Evaluation includes the impact of sequencing platforms, sample sizes, data structures, and the recapitulation of known degrees of variability. Other than generic metrics, other metrics are designed for analyzing transcriptomic data and implemented following package tutorials. All metrics can be processed through the wrapper method scVar [17]

 In this study, 14 commonly used metrics to quantify gene expression variability represent four distinct categories: generic metrics, local normalization metrics, regression-based metrics, and Bayesian-based metrics were included (Fig. 1; see the “Methods” section for details). These metrics are either generic or specifically designed in a package for analyzing transcriptomic data. We used log CPM-normalized gene expression as input for the generic metrics and local normalization metrics categories, and raw gene count matrix for the metric in other categories. One of these metrics, LCV, was originally designed for bulk RNA-seq data so we have modified it to be compatible with assumptions for scRNA-seq data. Unless stated, the metrics were run with default parameters as described in their respective vignettes and papers on a combination of simulated and real biological datasets (Table 1).

**Table 1:** Detailed summary of the datasets used for comparing metric performance

**Name**	**Platform**	**#Cells**	**#Genes**	**Cell type**	**Reference**	**Accessibility/data source**
HSC_droplet	3′-end (10X Droplet)	486	20,138	Hematopoietic precursor cell	[11]	[18]
Naive B_droplet	3′-end (10X Droplet)	49	20,138	Naïve B cell		
HSC_FACS	Full-length (smartseq2)	1174	22,899	Hematopoietic stem cell		
Naive B_FACS	Full-length (smartseq2)	1166	22,899	Naïve B cell		
Immature B_FACS	Full-length (smartseq2)	44	22,899	Immature B cell		
Large B	3′-end (10X Droplet)	10,085	15,858	CD19 + B cell	[19]	^a^SRA: SRP073767 [20]
Technical data	3′-end (10X Droplet)	1015	92	ERCC spike-in	[21]	GEO: GSE54695 [22]
Simulation1	Splatter Parameter estimated from full-length (10X Droplet)	400	1000		[23]	[24, 25]
Simulation2		400	1000			
Simulation3		400	1000			
Simulation4		400	1000			

^a^ http://support.10xgenomics.com/single-cell/datasets

### Estimating biological variability from scRNA-seq data is influenced by a dataset’s structure

Statistics often perform in a data-dependent manner and the ability to measure biological variability from scRNA-seq data is no exception. Different statistical metrics have been proposed to estimate biological variability and each metric comes with its own strengths and weaknesses. Since these metrics first appeared, the generation of scRNA-seq data and the associated analytical approaches have matured. Collectively, we understand that while datasets can vary widely, there are some structures that scRNA-seq datasets share. This section of our study investigates how specific kinds of biological and data-specific features impact different metrics for estimating biological variability and aims to identify which metric has the best overall performance.

#### Investigating how sequencing methods impact metric performance

**Fig. 2:**
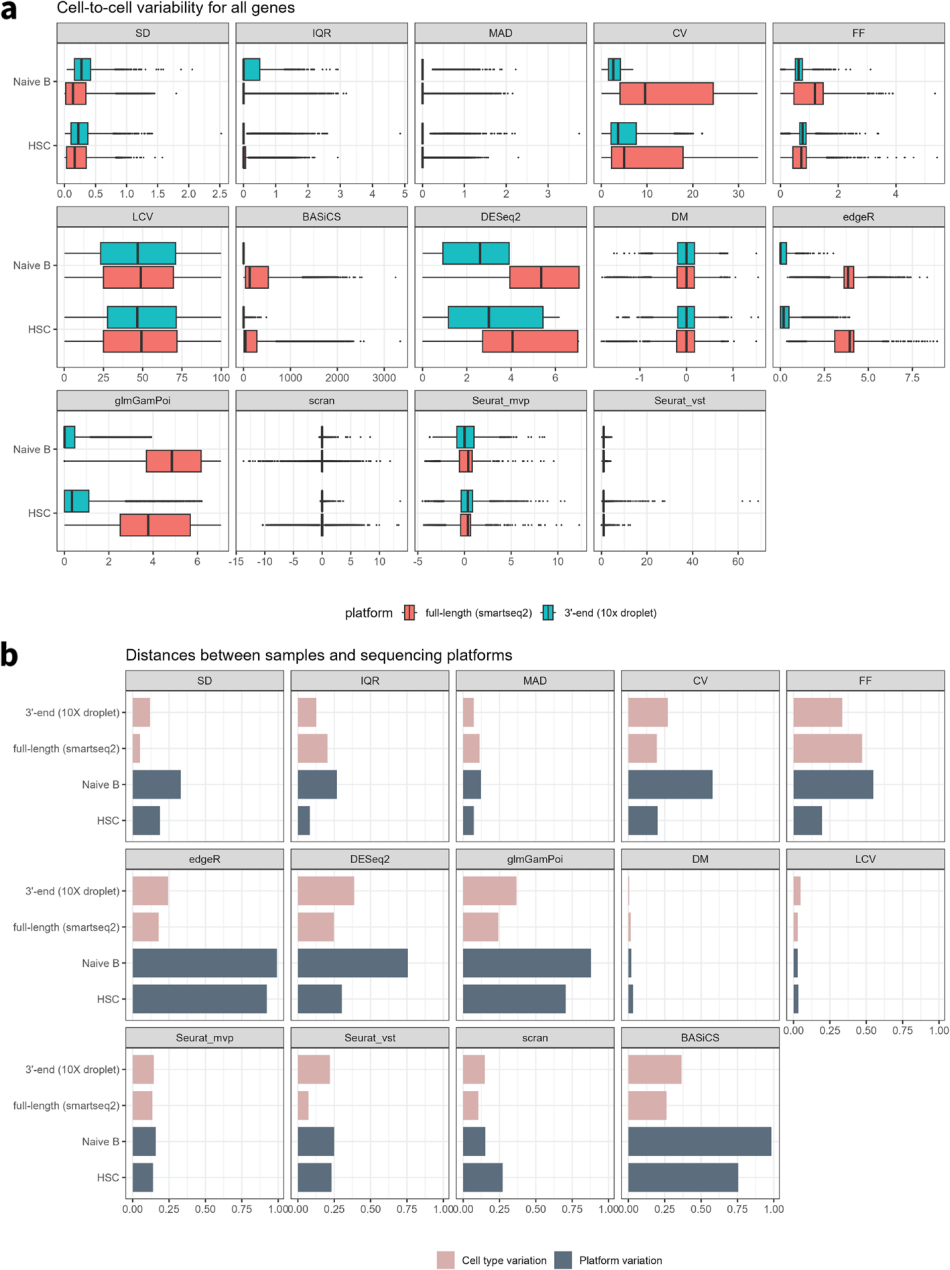
Cell-to-cell variability for all genes was measured from 14 metrics between two cell types with two sequencing platforms. **a** Boxplots for the cell-to-cell variability in each unique sample, colored by the sequencing platform. **b** Barplot demonstrated the distance calculated from Kolmogorov–Smirnov’s D statistic between the same cell types that had been sequenced by two platforms (HSC and naïve B) as well as between two cell types under the same sequencing platforms (full-length (smartseq2) and 3′-end (10X droplet))

We assume that a reliable metric for measuring biological variability will estimate this parameter regardless of the sequencing platform used to generate the data. The design of the TMS dataset provided an opportunity to assess how metrics performed under two different sequencing platforms because the same cell types were sequenced with a full-length FACS-sorted Smartseq2 and a 3′-end 10X Genomics Droplet-based method. These two sequencing methods distinctly capture full-length or 3′ reads, resulting in unique data structures. Our results demonstrated that in general, the platform-specific differences in gene expression variability tend to be larger than the differences due to cell type. These comparisons indicate that platform effects are important to consider when measuring gene expression variability (Fig. 2a).

To quantitatively investigate the platform-specific effect, the Kolmogorov–Smirnov’s D statistic was used to measure the distance between the variability metric distributions between the two sequencing methods for the same cell type. We compared these values to the distance between different cells sequenced by the same sequencing method to evaluate the size of the platform-specific effect (Fig. 2b and Additional file 1: Table S1). For a well-performed metric, the distance between different sequencing platforms on the same cell type should be substantially lower than the distance between cell types sequenced by the same platform. In general, CV, DESeq2, edgeR, and glmGamPoi were impacted most significantly by sequencing methods for both cell types while DM, LCV, scran, and Seurat metrics were more robust and showed similar estimated variability within the same cell types regardless of the sequencing method.

It is important to recognize that all sequencing methods have their own degree of technical variability, and in fact, each data set comes with its own distribution of expression variability as estimated by each metric. This can be observed through the fact that the distance between the two sequencing methods for the same cell type was not zero for any of the comparisons made in this study (Additional file 1: Table S1). Therefore, the degree of overall variability even for the same cell type should be handled carefully when analyzing data from different sequencing methods.

#### Investigating the impact of sample size on metric performance

**Fig. 3:**
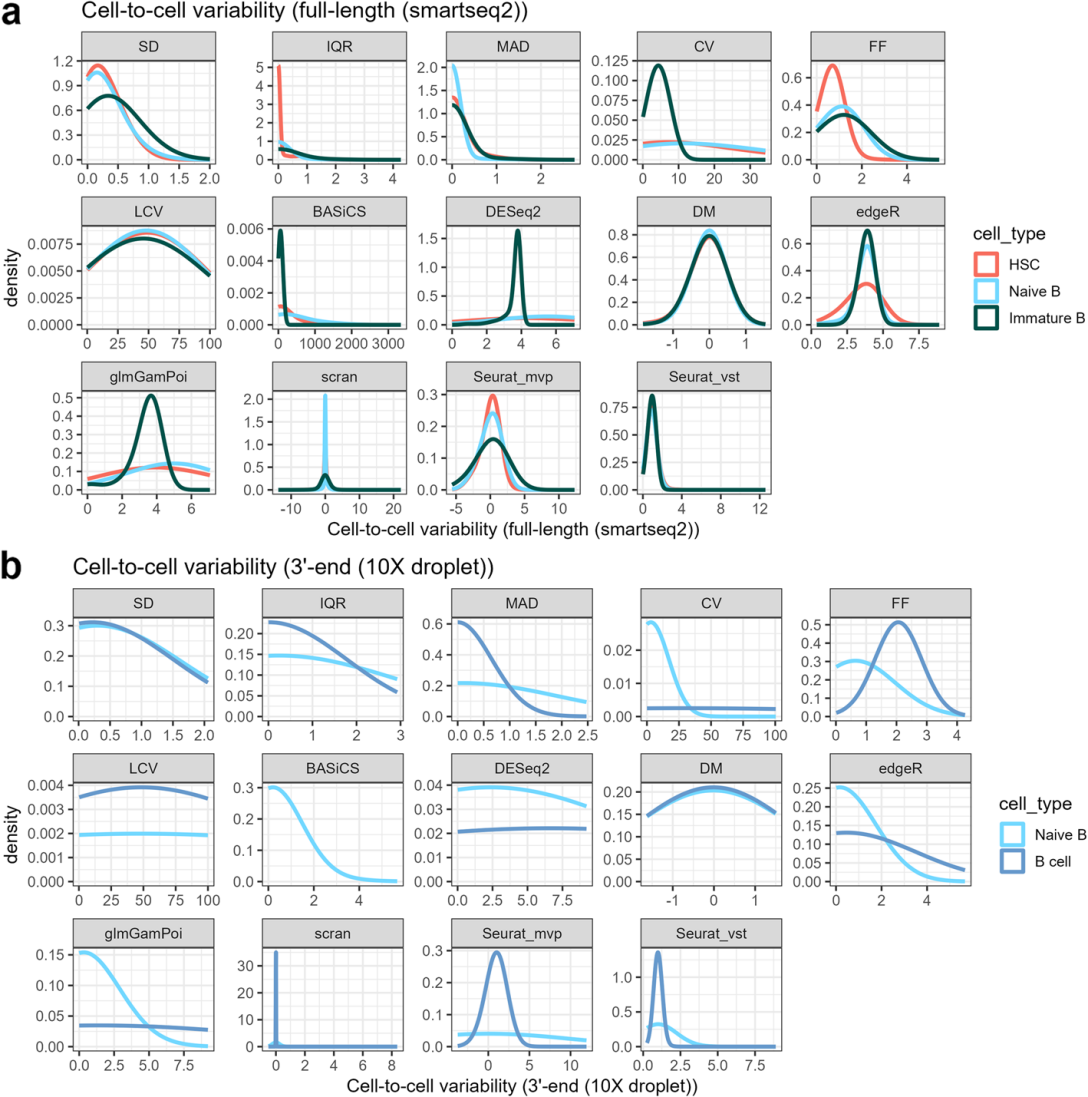
Cell-to-cell variability for all genes measured from the 14 metrics among several cell types with different sample sizes on the same sequencing platform. **a** Boxplots for the cell-to-cell variability in each unique sample, colored by sample sizes. **b** Boxplots for cell-to-cell variability for B cells from TMS and an external PBMC sample. *BASiCS was not applied to ultra-large B cells due to its computational complexity

Increasing the sample size tends to reduce the variance in a population and we investigated metric performance for scRNA-seq data with respect to this criterion. We used the number of cells available for different cell types under the same sequencing method to evaluate the impact of sample size on measuring cell-to-cell gene expression variability, e.g., HSC and naïve B (NB) cells each had a relatively large sample size (~ 1000 cells) versus immature B (IB) cells, which had only 50 cells in the TMS dataset (where the cell type sample sizes range from 50 to 1000 cells). Data with small sample sizes (~ 50 cells) showed a smaller range in their cell-to-cell variability distribution, especially when using metrics like CV, DESeq2, and edgeR (Fig. 3a). To further investigate the impact of sample sizes on the same cell type, we subsampled HSCs sequenced by two platforms from 10 to 90% of the total cell number and compared them with respect to full data. We found the distance between sub-sampled and full data decreased with the increasing number of sample sizes and reached a steady state at 50% where DESeq2, glmGamPoi, and CV showed the greatest distance which indicates these metrics were the most influenced by the reduction in cell sample size (Additional file 1: Fig. S1). In contrast, other metrics demonstrated substantial variation among replicates under the same sub-sampled sizes, especially for edgeR. Therefore, an insufficient number of cells may lead to biases when calculating the gene expression variability regardless of the metric chosen, resulting in underestimating the amount of cellular heterogeneity, especially in situation of rare cell types.

We also investigated the impact of sample size on the variability metrics using an external set of B cell ultra-large human datasets with more than 10k cells (10X droplet-based) [19]. The 10k B cell data was compared to the B cell (naïve B cell; 50 cells) in TMS droplet-based data (Fig. 3b). Overall, SD, IQR, scran, Seurat, and BASiCS showed a reduction in the variability estimated for the 10k B cells compared to the TMS B cell dataset. Surprisingly, CV, FF, DESeq2, DM, and edgeR showed increased gene expression variability for the 10k B cells. It is important to note that while the comparisons with this ultra-large dataset suggest that for categories like mean expression and SEGs, the performance of some metrics, including scran, appears to be worse for the datasets with more cells, this trend in performance is likely attributed to the fact that there is a higher degree of donor variability in the human B cell ultra-large datasets than in the laboratory-based mice in the TMS B cell dataset. Moreover, the list of SEGs was derived from human datasets where the sample sizes were relatively smaller (average 400 cells per cell type) than the number of B cells used in this study and this difference may therefore affect the performance observed.

The higher average gene expression in the 10k B cells (reflective of expression captured for a higher number of cells) may explain the performance of the genes that showed increased expression variability, as these metrics largely relied on the mean expression during estimation. This result highlights how the same cell type sequenced by the same method but with different sample sizes can impact the estimation of gene expression variability (Fig. 3). This analysis identified scran and Seurat as having better performance for this criterion as they showed the least amount of change for the two sample sizes tested. In addition to these trends in average expression, it is important to recognize that factors such as donor variability and sample size can greatly influence the overall performance of the metrics for estimating variability.

#### Investigating the impact of different data structures and biological properties on metric performance

Measuring biological variability is challenging because of how scRNA-seq data is structured. For example, an excess of zeros, low average expression, and variability that is influenced by both technical and biological sources can be difficult to untangle, and these aspects all result in an increased amount of noise in scRNA-seq data compared to bulk-level data. Therefore, it is important to select the metric that measures “true” biological variability and is not driven by the noise that can be sourced back to these specific data structures alone.

**Fig. 4:**
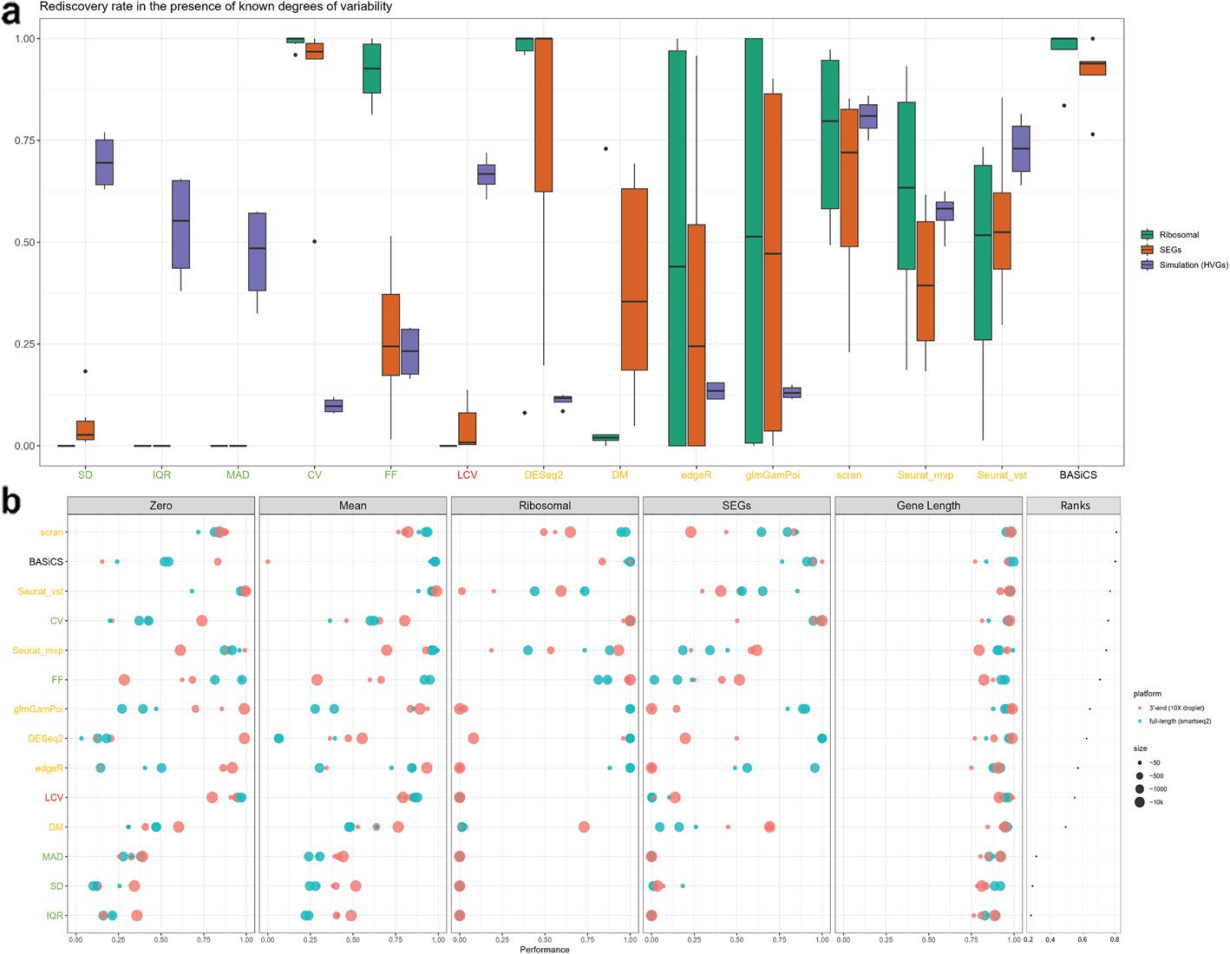
Metric performance in the presence of known degrees of variability and overall ranks for each metric. **a** Boxplots for the rediscovery rate for each metric based on ribosomal genes (ribosomal), stably expressed genes (SEGs), and simulations (HVGs). DM and BASiCS were not included due to missing gene length and batch information, respectively. **b** Bubble plot of metrics evaluation score across three cell types with two sequencing platforms. Each dot represented a dataset with its indicated number of cells. Each row represents a metric colored by one of the four metric categories. Each section represents one main criterion, including proportions of zero per gene (zero), mean expression (mean), ribosomal genes, SEGs, and gene length. The higher value indicates a stronger performance score where 1 indicates perfect performance

Our study investigated how different data structures influence the performance of the 14 metrics (Fig. 4 and Additional file 1: Fig. S2-S3) and found that scran and BASiCS performed well in most of the comparisons of the TMS cell-type data. We explored the influence of features like the number of zeros a gene has for all cells, the mean expression value, and the gene length. Although each metric handles zeros differently, we can still observe the increase in noise coming from the low signal genes in all the comparisons (Additional file 1: Fig. S3a). Additionally, most of the metrics preserved the mean–variance relationship except LCV, where lower average expression tends to correlate with a higher dispersion value (Additional file 1: Fig. S3b). Therefore, it is beneficial to pre-filter lowly expressed genes prior to any downstream analysis. Interestingly, even though the regression-based metrics and BASiCS considered the mean–variance relationship, the impact of data structures in these metrics showed diverse patterns. Gene length did not seem to impact the metrics when estimating gene expression variability (Additional file 1: Fig. S3c).

#### Investigating the impact of known degrees of variability on metric performance

To investigate the performance of the variability metrics under controlled settings, we generated two types of control datasets to include in our evaluation. Firstly, a negative control dataset was used where no biological variability was measured [21]. We measured the overall estimated variability distribution dispersion from each metric for this dataset. A lower dispersion value demonstrated the capability of a metric to detect biological variability rather than the total variability. As a result (Additional file 1: Fig. S4), most of the basic statistics metrics performed well except for CV and DESeq2. The ranked-based approach for LCV could not be used for this comparison because the final estimated values always ranged from 1 to 100. Among the GLM-based metrics, scran performed best. Secondly, a set of four simulation studies under different parameters were used, where 200 genes were known to be highly variable. Metrics were assessed based on the rediscovery rate (Fig. 4a), and scran, Seurat_vst, and SD obtained on average 70% of the rediscovery rate while DM, edgeR, and DESeq2 only achieved around 20%. Additionally, we used different cell type mixtures with increased data complexity to evaluate the metric comparison [26] (see the “Methods” section). This test serves as a positive control to evaluate the performance of the metrics where high biological variation exists. As expected, most of the metrics showed increased or stable overall variability as the complexity of the cell mixtures increased. Metrics which exhibited the most substantial changes in variability were BASiCS, CV, edegR, DESeq2, and glmGamPoi (Additional file 1: Fig. S5).

We also evaluated the metric performance on biologically relevant gene sets like ribosomal genes and stably expressed genes (SEGs) [27], where there is an overall expectation that these genes will have more stable gene expression. The gene rediscovery rate reflects how many of these genes’ expression variability are ranked within the first quartile of the metric’s gene expression variability distribution, indicating relatively stable expression (see the “Methods” section). The metrics based on generic statistics generally performed poorly by having a low rediscovery rate (Fig. 4a and Additional file 1: Fig. S3d-S3e); however, CV was outstanding in its preservation of these biologically relevant genes. LCV showed a very poor estimation of variability even though it performed well in eliminating the dependency from data structures. Most of the metrics based on regression model metrics performed well in preserving the SEGs, except for edgeR. Notably, DM excluded most of the ribosomal genes and some SEGs within its internal pre-filtering step. BASiCS performed consistently well in measuring the biologically relevant SEGs, especially for the dataset collected from the droplet-based sequencing method. Taking into account the performance results based on both sets of control datasets and biologically-relevant gene sets, scran was the best-performing metric under these scenarios (Fig. 4b).

Both B cells and endothelial cells are types of abundant cells that can exist in multiple tissues, and we used this property to gain further insight into the performance of the best-performing metric, i.e., scran. Only tissues with more than 70 cells were included, resulting in 5 tissues for B cells and 7 tissues for endothelial cells. We hypothesized that the HVGs that were commonly detected between different tissues would be important for supporting cell type-specific roles while the HVGs unique to a tissue would support tissue-specific effects. To test whether scran was able to make this distinction, we selected the top 500 HVGs from scran and compared them to the top 500 HVGs from CV for each tissue-cell-type combination. As shown in Additional file 1: Fig. S6, the HVGs measured using scran showed much more significant overlap across tissues compared to CV. The HVG gene lists measured from CV showed greater similarities to a random gene list as both gene lists show non-significant overlaps among tissue origins, suggesting a lack of true signal. Additionally, we confirmed that the HVGs measured by scran that were commonly detected between different tissues included many important cell type-specific markers (*Cd19*, *Cd83*, *Cd24a*, *Cd72*, and *Cd48* for B cells; *Cd9*, *Tmem66*, and *Tmem204* for endothelial cells). This analysis further indicates scran’s strength in performance at estimating gene expression variability.

### Fluctuations in cell-to-cell variability help explain the complex B lymphocyte differentiation process

**Fig. 5:**
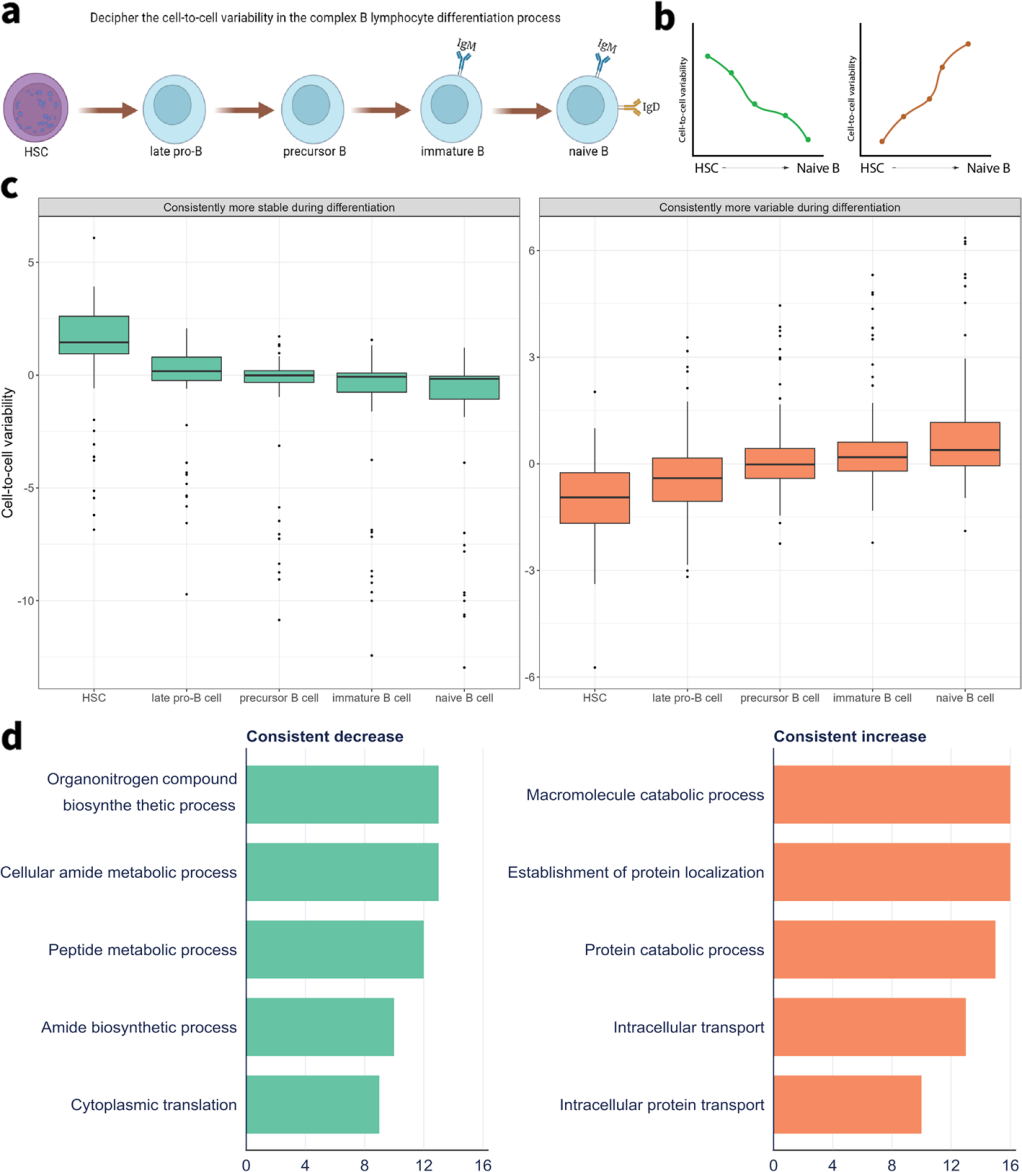
Deciphering the cell-to-cell variability of B lymphocytes during differentiation from TMS data. **a** Workflow illustration of the variability estimation from HSC to multiple B lymphocytes. **b** Line plot illustrating the two patterns of variability under study—increased stable and increased variable genes consistently during the differentiation process. **c** Box plots showed the estimated variability for identified genes for different cell types. **d** Bar plots showed the top 5 significant pathways related to the consistent decrease and increase genes with GO biological process terms. Green represents consistently stable, while orange represents consistently variable genes

Our comparative investigation into metric performance for estimating gene expression variability identified scran as the metric with the strongest overall performance. Hence, this metric was next applied to investigate how variability in cell-to-cell expression levels changed in HSC and B lymphocyte lineages and to identify specific markers of the differentiation process. We used the gene expression data from FACS Smartseq2 TMS marrow tissue and only incorporated cell types that had at least 100 cells in either young or old age groups in the lineages (Additional file 1: Fig. S7). The cell types included were late progenitor-B, precursor B, immature B, and naïve B cells (Fig. 5a). Gene expression levels were adjusted for the age effect using a regression model, given that the precursor B cells showed an overall significant difference between young and old groups (Additional file 1: Fig. S8).

Cell proliferation normally occurs through cell division that is highly associated with the G2M phase in the cell cycle. As expected, we saw that most of the cells in HSC and progenitor B cells were in G2M and S phases based on a list of markers [28] where they were prepared to proliferate while the cells in other cell types remained in G1 phase (Additional file 1: Fig. S9). In general, B lymphocyte differentiation occurs in the bone marrow from HSC through a complex transcriptional process, resulting in various B cell types [29]. Therefore, we re-constructed the lineage trajectory with a preset start [30] at HSC and observed the major trajectory from late progenitor-B, precursor B, immature B to naïve B cells (Additional file 1: Fig. S10), which aligned with the underlying biology. TMS marrow data supported the differentiation process because the major trajectory reconstruction showed transitions from HSC, late progenitor-B, precursor B, immature B to naïve B cells.

Identifying genes with changes in gene expression variability that show consistent trends during the lineage transition may be an insightful way to identify new markers or regulators. For example, a gene that is consistently decreasing its expression variability may reflect a gene whose expression is increasingly stable as it transitions from HSC to naive B cells. On the other hand, genes that have consistently increasing expression variability may be related to cell type-specific states during the differentiation processes [31].

We assessed the cell-to-cell variability changes along with the B lymphocyte lineages by identifying two gene expression patterns that were consistently more variable or stable in the differentiation process (Fig. 5b). Each pattern consisted of 89 consistently variable genes as well as 47 consistently stable genes (Fig. 5c). On average, there was an increased number of variable genes along the differentiation process relative to the stable genes. The top five markers for two patterns were *Ccr7*, *Tnfrsf13c*, *Cd19*, *Grap*, and *Itm2b* for the consistently variable genes, as well as *Oaz1*, *Rpl31*, *Rpl38*, *Rpl28*, and *Ccl9* for the consistently stable genes (Additional file 1: Fig. S11a-S11b).

Three cell differentiation markers, *Ccr7*, *Tnfrsf13c*, *Cd19*, showed the greatest variability changes along the differentiation process. Studies showed that these markers play essential roles in regulating immune-cell trafficking [32], B cell survival [33], and the B cell developmental process [34]. Furthermore, *Grap* helps *Erk MAP kinase* activation by connecting the B cell antigen receptor, which provides signal communications between a surface receptor to the DNA in the nucleus [35, 36], whereas *Itm2b* is known as a target of B cell lymphoma 6 protein repression [37]. Interestingly, these markers showed inconsistent degrees of average gene expression changes that defied the differentiation pattern, especially in HSCs. This result highlights the complementary information that cell-to-cell variability contributes towards identifying regulators of a differentiation process (Additional file 1: Table S2 and Fig. S11c). For example, *Tnfrsf13c* showed high but non-specific average gene expression between the four types of B cells whereas the level of cell-to-cell variability for this same gene reflected high specificity in naïve B cells. The genes that showed increased variability during cell differentiation belonged to pathways that were involved in B cell maturation. Conversely, most of the genes that were consistently more stable include the housekeeping gene *Oaz1* and ribosomal genes (*Rpl31*, *Rpl38*, and *Rpl28*). These genes are typically required for the maintenance of basic cellular functions and also do not demonstrate significant average expression changes among cell types (Additional file 1: Table S2).

The consistently variable genes showed more lymphoid-related specificity based on the percentage of expressed cells while consistently stable genes showed more generalized expression across different cell types (Additional file 1: Fig. S11d). Additionally, we performed pathway over-representation analysis of the two groups of genes to investigate their potential roles in B cell differentiation. Pathway analysis of increased variability genes showed a strong relationship with nitric oxide in the immune response whereas stably expressed genes were more strongly associated with ribosome and peptide metabolic process (Fig. 5d and Additional file 1: Table S3).

### Investigating cell-to-cell variability changes during aging in HSC and naïve B cells

**Fig. 6:**
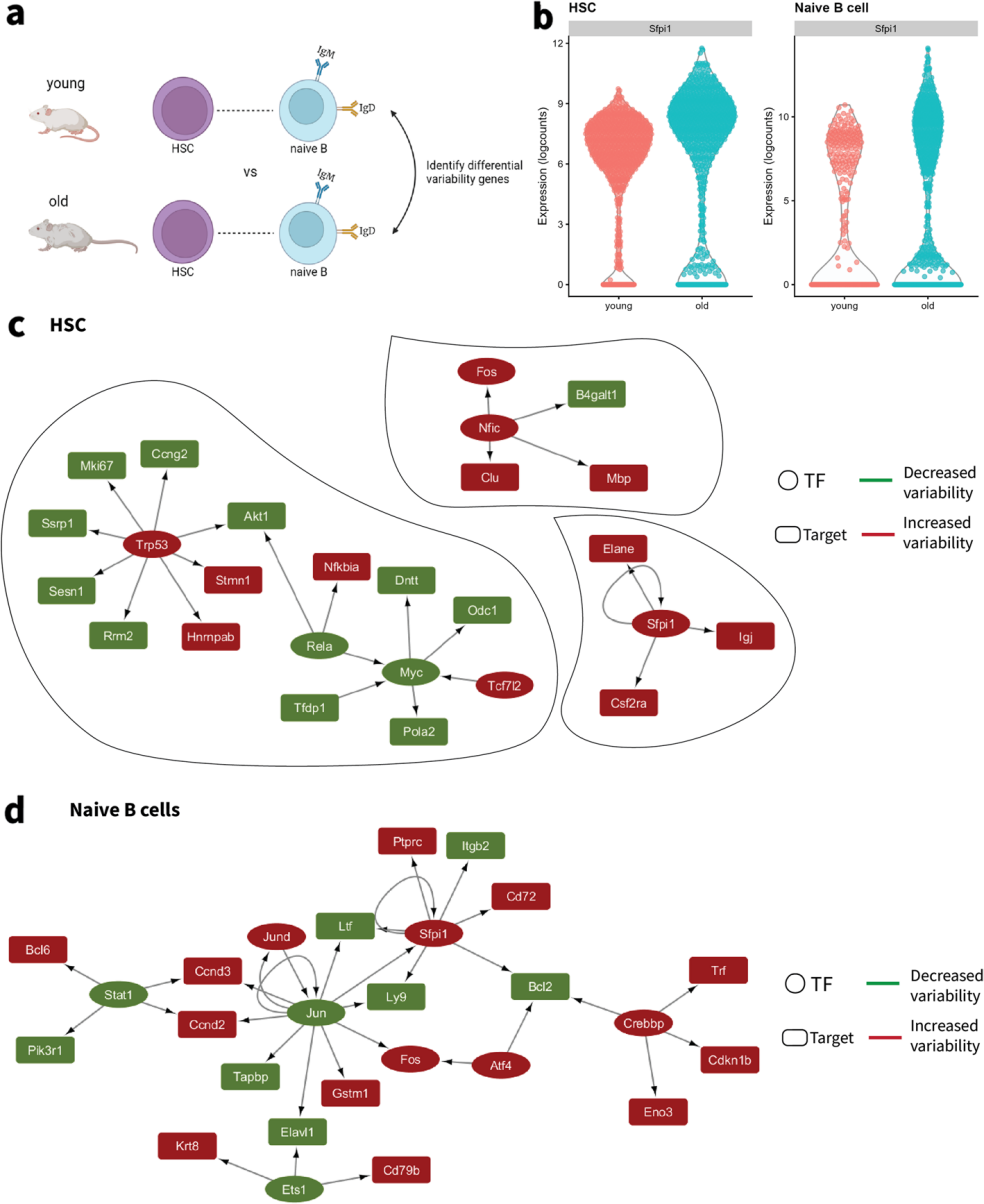
Deciphering the cell-to-cell variability of aging in HSCs and naïve B cells from TMS data. **a** Workflow illustration of the differentially variable analysis between young and old HSC and naïve B cells. **b** Violin plots shows Sfpi1 expression in young and old HSCs and NB groups with increased cell-to-cell variability in the old group compared to the young group. TF networks were identified for **c** HSC and **d** naïve B cells based on the differentially variable genes. A square represents a TF while an oval represents its target. Green represents decreased variability in the old compared to the young group and red represents increased variability

To decipher transcriptional heterogeneity changes in aging, we measured the cell-to-cell variability for HSC and naïve B cells in the young and old groups, respectively (Fig. 6a) using scran. We denote these genes as differentially variable (DV) to make the distinction from genes that are differentially expressed between two groups, in this case, young versus old. The DV genes can be interpreted as those genes with a statistically significant change in the variability of gene expression that is age-specific. We used these datasets to investigate how aging impacts the variability of gene expression in these two functionally relevant cell types, HSCs and naïve B cells. Overall, there was a slight decrease in variability observed in old HSC compared to young HSC (Wilcoxon test, *P*-value = 0.04) and no significant effect in naïve B cells (Wilcoxon test, *P*-value = 0.7, Additional file 1: Fig. S12b). This result aligns with the expectation of stem cell exhaustion and a decline in B lymphopoiesis with aging [38]. Genes that were highly variable in the young group versus the old group were more prevalent in both cell types (369 in HSCs, 327 in naive B cells). In contrast, there were 220 genes for the HSC and 269 genes for naïve B cells that were more variable in the old group versus the young group. We further compared the DV genes in aged HSCs from TMS data to an external dataset that contains sorted long-term HSCs (LT-HSCs) and short-term HSCs (ST-HSCs) [39]. The significant overlaps between DV genes from TMS data and two types of HSCs indicate the robustness of the markers across datasets (hypergeometric test, *P*-value < 0.05; Additional file 1: Fig. S12b-S12c). Interestingly, we identified a higher number of DV genes in aging ST-HSCs with respect to LT-HSCs supporting the prompt differentiation function in ST-HSCs but quiescent states in LT-HSCs [40]. Additionally, we examined whether DV genes identified in TMS experienced differential expression (DE) in terms of mean expression between the young and old groups. While the number of DE genes was substantially higher than the number of DV genes, we found at least 20% unique DV genes with no significant mean differences during aging in HSCs and naïve B cells (Additional file 1: Fig. S12d-S12e).

Assessing the roles of DV genes in biological processes using pathway enrichment analysis, we found that most enriched pathways were associated with the genes that reduced cell-to-cell variability in both cell types. Some shared pathways like ribosome and COVID-19 pathways may reflect the fluctuations occurring in tightened protein synthesis in aging. In addition, DNA replication and cell cycle pathways are enriched explicitly for the aging HSC, which may explain the reduced activation of proliferation from a quiescence state to sustain hematopoiesis [41]. On the other hand, pathways like oxidative phosphorylation that are enriched for the aging of naïve B cells may present the loss of functions for cell energy and proliferation [42] (Additional file 1: Fig. S12f).

The development and proliferation of progenitor cells into lymphoid and myeloid cell lineages are tightly controlled by transcription regulation networks [29]. To assess alterations of the transcriptional regulation, we constructed regulatory networks based on the genes that were significantly differentially variable between young and old groups, which have been identified as transcription factors (TFs) and their known TF targets [43]. The functions of the networks were highly associated with stem cell differentiation and cell maintenance. Interestingly, the analyses resulted in one connected network in naïve B cells and three smaller non-connected networks in HSC (Fig. 6c, d). Most DV genes that act as TFs have shown an increase in gene expression variability in older cells which may explain the dysregulation of regulatory mechanisms in cell type-specific aging. Additionally, we identified a shared TF, *Sfpi1*, demonstrating consistently increased variability in aging for both cell types (Fig. 6b) that was independent of the mean expression difference. However, the targets associated with *Sfpi1* presented unique expression changes for HSC and naïve B cells. *Sfpi1* is known as one of the primary lineage determinants that guide the multi-potential progenitors to establish a low-level expression of a mixed lineage pattern of gene expression, therefore favored in lineage-specificity [44]. Increasing variable *Sfpi1* expression led to more variable targets’ expression such as *Csf2ra*, *Elane*, and *Igj* in HSC while resulting in more stable expression of targets like *Bcl2*, *Ltf*, *Ly9*, *Itgb2*, and *Ptprc* in naïve B cells. Surprisingly, several targets showed significant mean gene expression differences between young and old groups regulated by fluctuated *Sfpi1* expression, emphasizing the influence of gene expression variability on controlling regulation which may be overlooked by only looking for mean gene expression changes (*Igj* and *Elane* in HSC; *Ptprc*, *Bcl2*, *Ly9*, and *Cd72* in naïve B cells; adjusted *P*-value < 0.01; Additional file 1: Fig. S12g).

## Discussion

Measures of cell-to-cell expression variability have been used extensively in the analysis of scRNA-seq data to understand heterogeneity and are often included in feature selection and dimension reduction steps. However, the application of cell-to-cell variability measurements should not be restricted to selecting highly variable genes but incorporating the investigation of gene expression variability changes within and between conditions. Subtle changes in cell-to-cell variability for a single gene may be helpful for detecting shifts in gene expression heterogeneity, especially during differentiation processes or aging. Studying cell-to-cell variability at the cell type level provides an interpretation of how gene expression variability is influencing regulation more directly than without acknowledging cell type groupings or modeling at the bulk level. There are tools such as MDSeq that use a novel re-parametrization of the negative binomial to provide flexible GLMs on gene expression variability analysis, and scDD that identify the distribution changes under four scenarios [45, 46]. However, these proposed methods focus on detecting the changes between conditions and not on estimating a single condition.

One study recently highlighted the need to account for variability to accurately determine differentially-expressed genes [47]. Failing to accurately account for such variability may lead to spurious biomarker identification that does not truly explain the underlying biology. But one of the greatest challenges in single-cell analysis is determining the best way to compute measures of variability. Here, we conducted a systematic evaluation of the metrics for gene expression variability and concluded that no metric performed consistently well in all criteria. Different classes of metrics had stronger performance with respect to specific criteria and this is likely to be a result of the limitations of the underlying algorithm. Our benchmarking analysis demonstrated that the widely used metric CV might introduce artificial variation due to the low mean expression resulting from excessive zeros in scRNA-seq data, especially in 10X droplet-based data (Additional file 1: Fig. S13). However, CV performed accurately at capturing expression variability for ribosomal genes and SEGs which indicated potential utility for stably expressed genes those with relatively low dropout. In fact, a novel method Cepo that identified differential stability was developed based on relative a CV metric [48]. Overall, our results demonstrated that most of the regression-model-based methods outperformed other metric categories which suggests that accounting for mean dependency in measuring gene expression variability is important.

Our study identified markers that had variable expression in the HSC to B lymphocytes differentiation process in bone marrow data. These types of variation changes that are continuous and subtle may mimic the continuous proliferation and differentiation processes for stem cells. Modeling consistent trends in increasing gene expression variability during the B lymphoid differentiation process identified not only genes that were known B cell maturation markers but also markers that control communication of signals through B cell antigen receptor connections. These consistently increasing expression genes represented the active cell fate decisions along with the differentiation, which is vitally important to autoimmunity and immunodeficiency [49]. Conversely, we identified *Oaz1*, which is known as a negative regulator of cell proliferation, together with other known ribosomal housekeeping genes that showed the most significant patterns in decreasing variability as cells transition from HSCs to B lymphocytes.

The role of transcription factors in modulating cell-to-cell variability can be useful for understanding how heterogeneity affects regulatory networks in aging. We identified genes that were differentially variable between old and young mice, and we used these genes as input to construct regulatory networks based on the TFs for HSC and naïve B cells. Our results identified a shared TF *Sfpi1* that was consistently variable for both HSC and naïve B cells networks and has been identified as a key dosage-dependent regulator of several hematopoietic cell lineage development and is associated with cell fate decisions [50]. Interestingly, *Sfpi1* also targeted a more variable *Cd72* in aged naïve B cells, in which *Cd72* is a primary regulator that appears to mediate B-cell and T-cell interaction. Moreover, differentially variable *Sfpi1* targets were distinct for HSC and naive B cells and their direction of expression changes also differed. The cell type-specific regulatory networks from our studies identified the unique TF-target interplay changes during the aging process, which may provide novel targets to predict the consequences of aging in HSC and B cells.

There are multiple reports of increased cell-to-cell variability with aging in different cell types. However, some studies have also reported uncertain or inconsistent levels of cell-to-cell variability changes in aging by similar experimental settings. For example, Marti et al. applied a quantitative statistical model on TMS and found sets of genes showed a robust decrease of noise with age [51]. One reason could be the biological differences in aging processes among different species, organs, and conditions, leading to the hypothesis that the increased transcription noise in aging appears to be gene-specific rather than tissue-specific. In fact, such hypotheses have been validated in many studies especially for HSC [15, 39, 52]. For example, Kowalczyk et al*.* revealed that gene expression variability among HSC was predominantly associated with cell cycle and a decrease in differentiation ability during aging. On the other hand, it is also possible that the metrics are misused in estimating gene expression variability. With an increasing prevalence of multi-source and multi-condition datasets, the underlying data structures should be carefully considered before applying any metric to measure gene expression variability. Overall, our analysis provides evidence for applying model-based metrics like scran to accurately estimate biologically-relevant gene expression variability where we demonstrate its ability to provide novel insight into complex conditions, like differentiation and aging.

## Conclusions

Cell-to-cell variability is important for understanding cellular heterogeneity under different biological conditions. Here, we have performed a comprehensive benchmarking study to explore the performance of 14 metrics that quantify cell-to-cell variability. Based on the evaluation, we found that the gene expression variability estimates from the scran R package have the best performance in recapitulating the biological cell-to-cell variability and are independent of the data structures. We show the significant impact of different levels of cell-to-cell variability under two important biological processes such as differentiation and aging. Our analyses demonstrate that cell-to-cell variability changes reveal critical roles in not only maintaining cell functions but also accurately capturing the key regulators. We conclude that cell-to-cell variability should not primarily be considered as noise in analyzing scRNA-seq data, but also as a statistic that provides remarkable information for understanding complex biological processes.

## Methods

### Overview of metrics

We examined 14 metrics that are currently available to measure gene expression variability in transcriptome data. Five of them are specially designed for analyzing scRNA-seq data (DM, glmGamPoi, scran, Seurat, and BASiCS), with more details in Table 2. Scater [53] is applied to normalize the raw data if needed.

**Table 2:** Metrics information summary

**Metric**	**Category**	**Input type**	**Package Version**	**Reference**
MAD	Generic	Normalized	stats version 4.1.1	-
SD	Generic	Normalized	stats version 4.1.1	-
IQR	Generic	Normalized	stats version 4.1.1	-
Coefficient of variation	Generic	Normalized	scVar version 1.0.0	[17, 54]
Fano Factor	Generic	Normalized	scVar version 1.0.0	[17, 54]
Local coefficient of variation	Local normalization	Normalized	scVar version 1.0.0	[55]
DESeq2	Regression-model based	Raw	DESeq2 version 1.32.0	[56]
Distance to median (DM)	Regression-model based	Raw	scVar version 1.0.0	[17, 54]
edgeR	Regression-model based	Raw	edgeR version 3.34.1	[57]
glmGamPoi	Regression-model based	Raw	glmGamPoi version 1.4.0	[58]
scran	Regression-model based	Raw	scran version 1.20.1	[59]
Seurat_mvp	Regression-model based	Raw	Seurat version 4.3.0	[60, 61]
Seurat_vst	Regression-model based	Raw	Seurat version 4.3.0	[60, 61]
BASiCS	Bayesian modeling	Raw	BASiCS version 2.4.0	[62]

#### Generic metrics

The five metrics in this category include median absolute deviation (MAD), interquartile range (IQR), standard deviation (SD), coefficient of variation (CV), and the Fano Factor (FF). SD, MAD, and IQR have been interpreted as a stochastic disturbance in the data which represents the uncertainty around the mean value, and have previously been applied to gene expression analyses [63]. Additionally, gene expression variability is known to show some dependence on the mean expression level, and the metrics CV (σ^2^/μ) and FF (σ/μ) are two of the most commonly used metrics for modeling variability in scRNA-seq data that explicitly acknowledge a mean–variance dependency [44, 45].

#### Local normalization metrics

The local coefficient of variation (*LCV*) [55] was first developed to rank the expression variability of each gene relative to genes with similar local expression values in bulk RNA-seq data. In our adaptation to scRNA-seq data, we have tried to retain as much of the ranking algorithm and the relationship between the mean and CV. LCV starts with ordering each gene according to its mean expression and then assigns its corresponding CV into the user-defined width. It re-calculates the local CV as the quantiles of the CV within the window for each gene. In such a way, this metric rescales variation within the whole gene population to a user-defined range.

#### Regression-model based metrics

Novel model-based approaches have been introduced to regress unwanted technical variation, to improve the accuracy and detection of “true” biological variation in the gene expression data. More importantly, these metrics apply different regression models to account for the mean–variance relationship and residuals are commonly counted as the cell-to-cell variability measurements. Therefore, genes with negative values represent biological variations that are lower than the expected values and vice versa. Several metrics in this category were included in our comparative study because of the different model assumptions that they adopt.

*DESeq2* [56] estimates dispersion by applying a generalized linear model of the form can be summarized as:

$${K}_{ij} \sim NB({\mu }_{ij},{\alpha }_{i})$$

where the raw count matrix ${K}_{ij}$ for gene $i$, sample $j$ are modeled by a negative binomial distribution with fitted mean ${\mu }_{ij}$ and a gene-specific dispersion parameter ${\alpha }_{i}$. ${\alpha }_{i}$ reflects the relationship between the normalized mean expression and the variance for the observed gene $i$ expression which is assumed to follow a log-normal prior distribution. Next, a curve is fitted to all gene-wise dispersions and further shrunk towards the expected dispersion value. The final dispersion estimates were used to represent gene expression variability.

*DM* (distance to the median): Kolodziejczyk et al. [64] developed a novel method to account for the confounding factor of expression levels when calculating cell-to-cell variability. The distance between the CV^2^ of each gene with a running median was calculated and then corrected for using the gene length to eliminate the confounding effect. They refer to this expression-level normalized measure of gene expression heterogeneity as DM.

*edgeR* [57] also models the gene counts using a negative binomial distribution and the estimation of the dispersion parameter was done in two steps. First, a common dispersion is estimated by maximizing the adjusted likelihood function on the squared biological coefficient of variation (BCV). These common dispersion estimates are further generalized by binning and weighting the subsets of the dispersion grid, so-called trended dispersion. Second, the weighted likelihood empirical Bayes is applied to squeeze the tagwise (gene-wise) dispersions towards a common dispersion by obtaining posterior dispersion estimates. These tagwise dispersions were used to estimate the gene expression variability in this comparative study.

*glmGamPoi* [58] leverages the inferior transformation approach, which outperforms DESeq2 and edgeR by substantially higher speed and the inference to be more suitable for scRNA-seq data by using efficient data representations. Through modeling under a Gamma-Poisson distribution, it also shows better estimates of the over-dispersion parameter on datasets with low counts.

*scran* [59] performs a unique normalization process by pooling cells to calculate multiple scaling factors, which leads to a more accurate estimate that improves downstream analysis. The total variance in expression for each gene is fitted with log-normalized expression by a mean–variance trend. To obtain an estimate of biological variability for each gene, the decomposition step is applied to the total variance by subtracting the fitted value (technical variability). The biological variability estimates were used to estimate gene expression variability in this comparison.

*Seurat* [60, 61] performs normalization by dividing gene counts for each cell by library size and further multiplying by 10,000. It includes two main methods to examine gene expression variability. One way is to use a binning method (default bin = 20) on the average expression and then calculate *z*-scores for dispersion within each bin for each gene (Seurat_mvp). This approach aims to control for the strong mean dependency when calculating the dispersion. Alternatively, it fits a trend line to the relationship of mean and variance using LOESS then standardizes and scales the feature values using the observed mean and expected variance (Seurat_vst). Both methods will be analyzed in the comparison separately.

#### Bayesian modeling metrics

*BASiCS* [62] is the first method that obtains estimates of biological variation using a Bayesian hierarchical model. The batch information was included for measuring technical variability in BASiCS. The biological variability is measured by a residual measure of variability given by the departures from a global mean/over-dispersion trend, with Regression = TRUE for both data types.

### Datasets and pre-processing used for metrics evaluation

To compare the performance of the different metrics, we focused on investigating changes in expression variability from the Tabula Muris Senis (TMS) data at the cell type level. TMS is a large publicly available mouse atlas that includes scRNA-seq data, generated from both FACS-sorted single cells and through droplet sequencing, across 24 organs with rich transcriptome information [11]. FACS data allow for higher sensitivity and less sparsity whereas droplet-based sequencing enables more cells to be analyzed. We downloaded the raw gene expression count matrix (FACS-sorted Smart-seq2 and 10X Genomics droplet-based) from the figshare repository (10.6084/m9.figshare.8273102.v3) [18] and normalized these data using the Seurat package (version 3) [65] following the descriptions given in the original paper. For our metric evaluation, only 24-month male mice were included for the downstream analysis. We estimated cell-to-cell variation exclusively at the level of a single cell type.

Marrow tissue was selected for its multipotent capabilities and involvement in the immune response. We also selected this tissue for statistical reasons because marrow tissue had the largest number of cells that were sequenced. Out of the 23 cell types in the marrow tissue, several cell types were carefully chosen due to the data properties, as summarized in Table 1. HSC and Naïve B cells were two of the most representative and abundant cell types that were sequenced in the full-length smartseq2 technology. Corresponding cell types that were sequenced by 10x droplet technology were also included as a comparison for the metric performance. The immature B cells were selected because they had a relatively smaller sample size and this is an important contrasting comparison to include because the sample size is critical in measuring gene expression variability estimation sensitivity [23]. In addition, we applied these metrics on B cells and endothelial cells from all tissues available in TMS to evaluate the metric’s ability to detect genes that are responsible for functional maintenance across tissues.

Beyond TMS, two external datasets were further tested as controls to investigate the metric performance. The first dataset was sequenced from 92 synthetic spike-in RNA molecules from the External RNA Controls Consortium (ERCC) to understand technical variability and was downloaded from NCBI Gene Expression Omnibus (GEO; http://www.ncbi.nlm.nih.gov/geo/) under accession number GSE54695 [21]. In theory, there is no genuine biological variability derived from this experiment, so this dataset represents a limited level of variability compared to other gene expression data. The second dataset was included because it has a much larger sample size, containing more than 10k sorted CD19 + B cells from fresh PBMCs which is 10 times greater than the TMS datasets, which can be downloaded from http://support.10xgenomics.com/single-cell/datasets [19]. As expression variability is reduced with sample size, this comparison demonstrates how well each matrix can handle big data.

To investigate the impact of sample size on metric performance, we sub-sampled HSCs sequenced by the FACS Smart-seq2 and 10X droplet technology. We down-sampled the data into 10%, 20%, 50%, 80% and 90%, with each procedure repeated 5 times. To evaluate the metric performance, we compared all gene distribution from each sub-sampled data against the full data distribution by Kolmogorov–Smirnov D statistics.

Although there were studies that identified stably expressed genes, it is challenging to identify the ground truth for highly variable genes with real data. Therefore, four independent simulation studies were generated from parameters that were comparable to the TMS dataset via the Bioconductor package Splatter [23, 24]. Simulation 1 (Sim1) and Simulation 2 (Sim2) consisted of 200 genes by setting the BCV parameters to 3-fold and 4-fold of the original parameter to imitate the different levels of variability. Additionally, to accommodate extremely noisy and sparse scRNA-seq data, we modified the dropout.mid from 2 to 10 on Sim1 and Sim2, denoted as Sim3 and Sim4. For each simulated dataset, the total number of cells was set to 400 and the total number of genes was set to 1000, where 200 of the genes were known to be highly variable.

Alternatively, we curated complex cell populations with different levels of data complexity to evaluate the metric performance with increased biological variability within the data. We used a combination of cell-types mixtures from CD4-positive, alpha–beta T cell, granulocytopoietic cell, precursor B cell, promonocyte, and granulocytes based on the sample correlation based on the average expression [26].

### Metrics evaluation score

To assess the performance of each metric, we measured the associations between each metric and characteristics of the data, such as the proportions of zeros, the mean expression, and the gene length. As DM required a pre-filtering step, it resulted in fewer genes available to be tested in the analysis (a loss of about 40% of genes) which affected its evaluation of performance in our comparison. Additionally, the log-transformed mean expression was used for all metrics, except for edgeR, DESeq2, Seurat, scran, DM, glmGamPoi, and BASiCS, as they have different internal normalization approaches that resulted in different mean expression values. Available gene length was retrieved for 18,000 genes from the mm9 reference by the goseq package (version 1.44.0) [66]. The degree of association was assessed by Pearson correlation coefficient and the corresponding *R*-value was kept as the measurement score. The higher *R*-value represented a stronger association between the estimated variability and other data characteristics, which reflected a greater influence on performance due to the data structures. For assessing the metric performance on genes that were expected to have low expression variability, we used ribosomal genes and mouse stably expressed genes (SEGs) [27] because they have housekeeping roles that are critical to maintaining the fundamental functions of the cell. We scored the metrics based on the number of genes from these categories (ribosomal genes or SEGs) that were in the first quartile of the estimated variability distribution for each metric. The higher proportion of genes that followed such criteria, the better performance of the metric because it accurately calculated the lowly variable genes. For the simulation data, the rediscovery rate was calculated to be determined by the number of identified HVG genes.

### TMS B cell studies and pre-processing

To understand how cell-to-cell variability changes in B cell development, we included the FACS-sequenced data from 3-month and 24-month-old mice as they have a relatively higher abundance of sequenced cells in TMS compared to other time points. We selected the B lineage-related cell types that have at least 100 cells in either age group, resulting in HSCs, late progenitor-B, precursor B, immature B (IB), and naïve B cells (NB). Raw data was processed according to descriptions in the original paper for all cell types and conditions. To increase the sample size for analysis of the variability patterns in HSC and B cell lineages, we merged both age groups for each cell type to regress out the age effect using a design matrix. Conversely, 3-month and 24-month HSC and naïve B cells were used for measuring differentially variable genes in aging. To increase the statistical power, only genes that expressed more than 10% of the total cell population were retained.

### Trajectory analysis

Pseudotime trajectory analysis was performed to determine how cells transition from one state to another based on the TMS B cell lineage datasets. We constructed the trajectory with Monocle3, which presets the starting point as HSC [30]. Cell pseudo-time values were applied and colored for each cell identity on the UMAP to indicate the potential trajectory along each developmental process.

### Network of transcriptional factors and target genes

RegNetwork [43] was used to retrieve the transcription factors and their target genes. TF networks were constructed using Cytoscape (v3.7.1) [67], where the arrows denote a link from a TF to a target gene. TFs were annotated with rectangles and targets were annotated with circles. The color indicates whether a gene was more variable or less variable in the old compared to the young group.

### Differentially Variable (DV) gene testing

To increase the power for calculating DV genes, we first pre-filtered the genes based on the proportions of zeros so that only genes that expressed more than 5% of the total cell population were retained. To determine the differentially variable genes, we calculated the cell-to-cell variability (CCV) difference δ measured by scran, which is defined as δ = CCV (Old) – CCV (Young). Then δ was normalized as a *z*-score and further converted to *p*-values with the assumption that all *z*-scores follow a normal distribution. In this paper, we selected the features whose *p*-value < 0.05.

### Differentially Expressed (DE) gene testing

To evaluate the average expression changes in aged HSCs and naïve B cells, DE analysis was applied using scran [59]. We used the same 5% threshold as the DV analysis to remove genes with high dropout with adjusted *p*-value < 0.01. We retained the same number of DE genes as DV genes in each cell type based on the log fold-change and compared using the upset plots [68].

### External HSC dataset to validate DV genes in TMS HSCs

To validate the DV genes identified in TMS HSCs, we used an external dataset with FACS-sorted long-term HSCs (LT-HSCs) and short-term HSCs (ST-HSCs) from young (8–12 weeks) and old (20–24 months) mice [39]. These pre-sorted HSCs were sequenced with full-length SMART-Seq2 sequencing protocols, which can be downloaded from NCBI Gene Expression Omnibus (GEO; http://www.ncbi.nlm.nih.gov/geo/) under accession number GSE100428. DV testing was applied on LT-HSCs and ST-HSCs separately between young and old groups under the same settings as the DV testing on TMS HSC. The significant test on the overlaps of genes in LT-HSCs and ST-HSCs with respect to TMS HSC was examined by a hypergeometric test in phyper R function.

### Pathway over-representation analysis

To investigate gene sets at the molecular and functional level, we performed enrichment analysis on GO biological processes pathways by using MSigDB [69] for identifying the biological functions of genes under B cell differentiation pattern and KEGG database by clusterProfiler [70] for revealing biological functions with respect to aged HSCs and naïve B cells, with all genes in TMS data as universe background. The top 5 significant pathways (adjusted *P*-value < 0.05) were shown in the barplot and ordered by their overlapped gene set size.

### Supplementary Information

**Additional file 1: Table S1.** Assessing differences due to cell type and sequencing platform. **Table S2.** Identifying the top consistently variable and stable genes along the B lymphocytes differentiation process. **Table S3.** Top Five Over-represented GO BP Terms for Consistently Variable and Stable Genes. **Fig. S1.** Investigating the impact of downsampling on expression variability metrics for different sequencing platforms. **Fig. S2.** Heatmaps of metrics performance on three cell types with two sequencing platforms. **Fig. S3.** Evaluating cell-to-cell variability metrics with respect to each data characteristic criteria. **Fig. S4.** Investigating the impact of cell-to-cell variability metrics for ERCC controls. **Fig. S5.** Metric performance in cell mixtures with different levels of data complexity. **Fig. S6.** Investigating the overlap between HVGs in B cells and endothelial cells that reside in different tissues. **Fig. S7.** Investigating the number of cells in marrow tissue in old and young groups from TMS that were sequenced by FACs-smartseq2. **Fig. S8.** Volinplot of gene expression variability for five cell types between young and old groups from Tabula-Muris-Senis data. **Fig. S9.** The cell cycle stages of HSC, late pro-B cells, precursor B cells, immature B cells and naïve B cells. **Fig. S10.** Pseudotime inferences for each cell type after removal of the age effect. **Fig. S11.** The top 5 consistently variable and stable genes along the B cell lymphocytes differentiation process. **Fig. S12.** Cell-to-cell variability alterations in HSC and B lymphopoiesis in aging. **Fig. S13.** Metric performance evaluated per sequencing platform.

**Additional file 2.** Review history.

## Data Availability

The datasets and metrics used in this analysis are all publicly available and described in the “Methods” section and Table 1 with all links. Codes to reproduce the presented analyses and figures are available at GitHub (https://github.com/huiwenzh/cell-to-cell-variability-changes-in-ageing) [71] and have been deposited to Zenodo via accession identifier (10.5281/zenodo.8210511) [25]. We also provide implementations of these 14 metrics compared in this work as scVar Github package (https://github.com/huiwenzh/scVar) [17] which have been deposited on Zenodo via accession identifier (10.5281/zenodo.8166143) [54]. The package and source codes are licensed under Creative Commons (CC) license. Statistical analysis was performed using R version 3.6 (https://www.r-project.org/). Plots were generated through the ggplot2 version 3.3.2 package.
