# Supplementary material for: Measuring cell-to-cell expression variability in single-cell RNA-sequencing data: a comparative analysis and applications to B cell aging
Source: Genome Biol. 2023 Oct 20;24:238. doi: 10.1186/s13059-023-03036-2 (PMC10588274; doi:10.1186/s13059-023-03036-2)
Supplement: Supplementary file 1 — Additional file 1: Table S1. Assessing differences due to cell type and sequencing platform. Table S2. Identifying the top consistently variable and stable genes along the B lymphocytes differentiation process. Table S3. Top Five Over-represented GO BP Terms for Consistently Variable and Stable Genes. Fig. S1. Investigating the impact of downsampling on expression variability metrics for different sequencing platforms. Fig. S2. Heatmaps of metrics performance on three cell types with two sequencing platforms. Fig. S3. Evaluating cell-to-cell variability metrics with respect to each data characteristic criteria. Fig. S4. Investigating the impact of cell-to-cell variability metrics for ERCC controls. Fig. S5. Metric performance in cell mixtures with different levels of data complexity. Fig. S6. Investigating the overlap between HVGs in B cells and endothelial cells that reside in different tissues. Fig. S7. Investigating the number of cells in marrow tissue in old and young groups from TMS that were sequenced by FACs-smartseq2. Fig. S8. Volinplot of gene expression variability for five cell types between young and old groups from Tabula-Muris-Senis data. Fig. S9. The cell cycle stages of HSC, late pro-B cells, precursor B cells, immature B cells and naïve B cells. Fig. S10. Pseudotime inferences for each cell type after removal of the age effect. Fig. S11. The top 5 consistently variable and stable genes along the B cell lymphocytes differentiation process. Fig. S12. Cell-to-cell variability alterations in HSC and B lymphopoiesis in aging. Fig. S13. Metric performance evaluated per sequencing platform. [file 13059_2023_3036_MOESM1_ESM.docx]

## **Supplementary – Tables**

Table S1: Kolmogorov–Smirnov’s D statistic measured the distribution difference between two sequencing platforms for the same cell types as well as the difference between two cell types for the same sequencing platforms. The distance highlighted in light blue represented the metric measured biological similarity rather than technical (platform) whereas yellow highlighted the opposite scenario.

|  | HSC_FACS vs HSC_droplet | HSC_FACS vs NB_ FACS | NB_FACS vs NB_droplet | HSC_droplet vs NB_droplet |
| --- | --- | --- | --- | --- |
| SD | 0.19 | 0.05 | 0.33 | 0.12 |
| IQR | 0.08 | 0.20 | 0.27 | 0.13 |
| MAD | 0.07 | 0.11 | 0.12 | 0.07 |
| CV | 0.20 | 0.20 | 0.58 | 0.27 |
| FF | 0.20 | 0.47 | 0.55 | 0.33 |
| edgeR | 0.92 | 0.18 | 0.99 | 0.24 |
| DESeq2 | 0.30 | 0.25 | 0.76 | 0.39 |
| DM | 0.03 | 0.02 | 0.02 | 0.01 |
| glmGamPoi | 0.70 | 0.24 | 0.88 | 0.37 |
| LCV | 0.03 | 0.03 | 0.03 | 0.05 |
| Seurat_mvp | 0.14 | 0.14 | 0.16 | 0.15 |
| Seurat_vst | 0.23 | 0.07 | 0.25 | 0.22 |
| scran | 0.27 | 0.10 | 0.15 | 0.15 |
| BASiCS | 0.75 | 0.26 | 0.98 | 0.37 |

Table S2: Differentially expressed information with respect to mean difference for the top consistently variable and stable genes along the B lymphocytes differentiation process. Differentially expressed genes were identified by scran with FDR values at 0.05. Up and down arrows represented such gene with significant increase and decrease in the cell type with respect to other cell types (1-vs-all fashion), respectively.

|  | **Top consistently variable genes** | | | | | **Top consistently stable genes** | | | | |
| --- | --- | --- | --- | --- | --- | --- | --- | --- | --- | --- |
|  | *Cd19* | *Tnfrs13c* | *Ccr7* | *Itm2b* | *Grap* | *Oaz1* | *Rpl31* | *Rpl38* | *Rpl28* | *CCl9* |
| **HSC** | 🡮 | 🡮 | 🡮 | 🡭 | 🡮 |  | 🡭 |  | 🡮 | 🡭 |
| **pro-B** |  |  |  |  | 🡭 | 🡭 |  | 🡭 |  |  |
| **pre-B** |  |  |  |  | 🡮 |  |  | 🡮 |  |  |
| **IB** |  | 🡭 |  |  |  |  |  |  |  |  |
| **NB** |  |  | 🡭 | 🡭 |  | 🡮 |  |  |  |  |

Table S3: Top 5 over-represented pathways for the consistently variable and consistently stable genes along the B lymphocytes differentiation process against GO biological process database.

~~
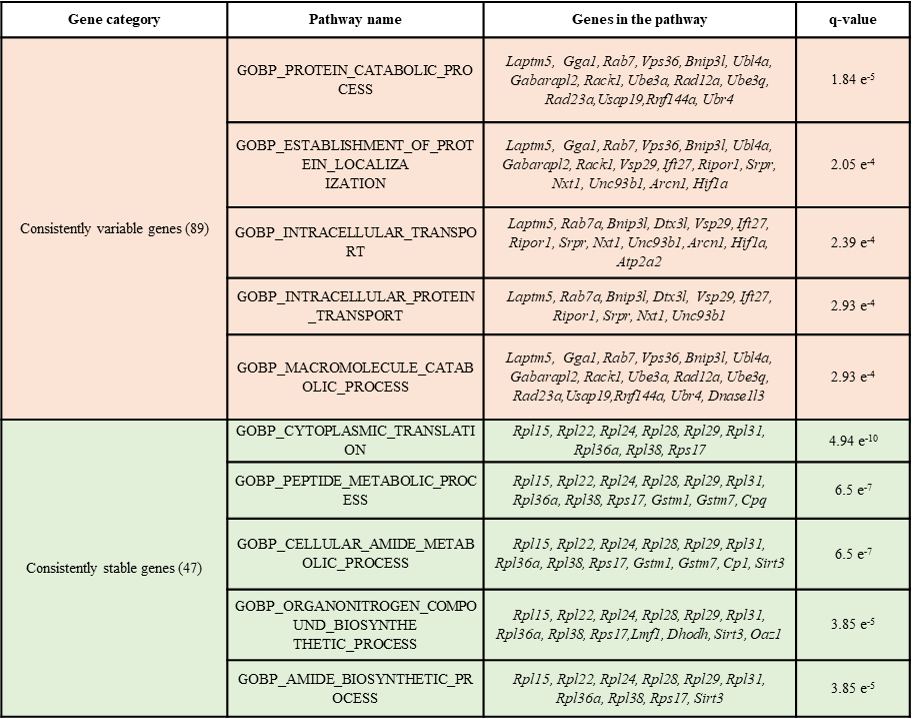
~~


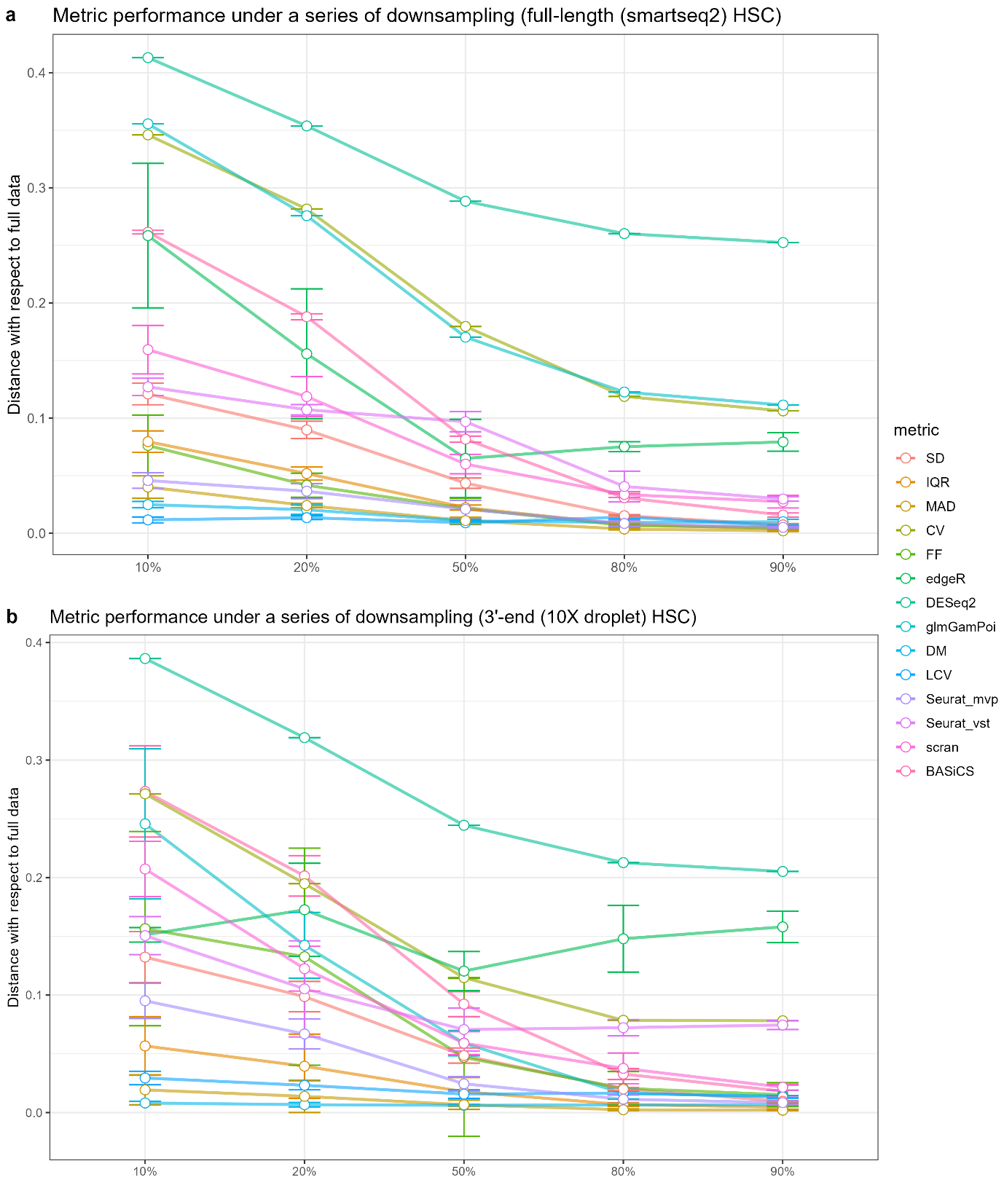


Fig. S1. Metric performance in the subsampled HSCs sequenced from a) full-length smartseq2 and b) 3’-end 10x droplet platforms, ranging from 10%, 20%, 50%, 80% and 90% downsampled to the full data. Distances between samples were calculated by Kolmogorov–Smirnov D statistic. Each line represented the metric used in the evaluation and the error bar represented the standard deviation of the distance calculated from the five replicates per subsampled data.


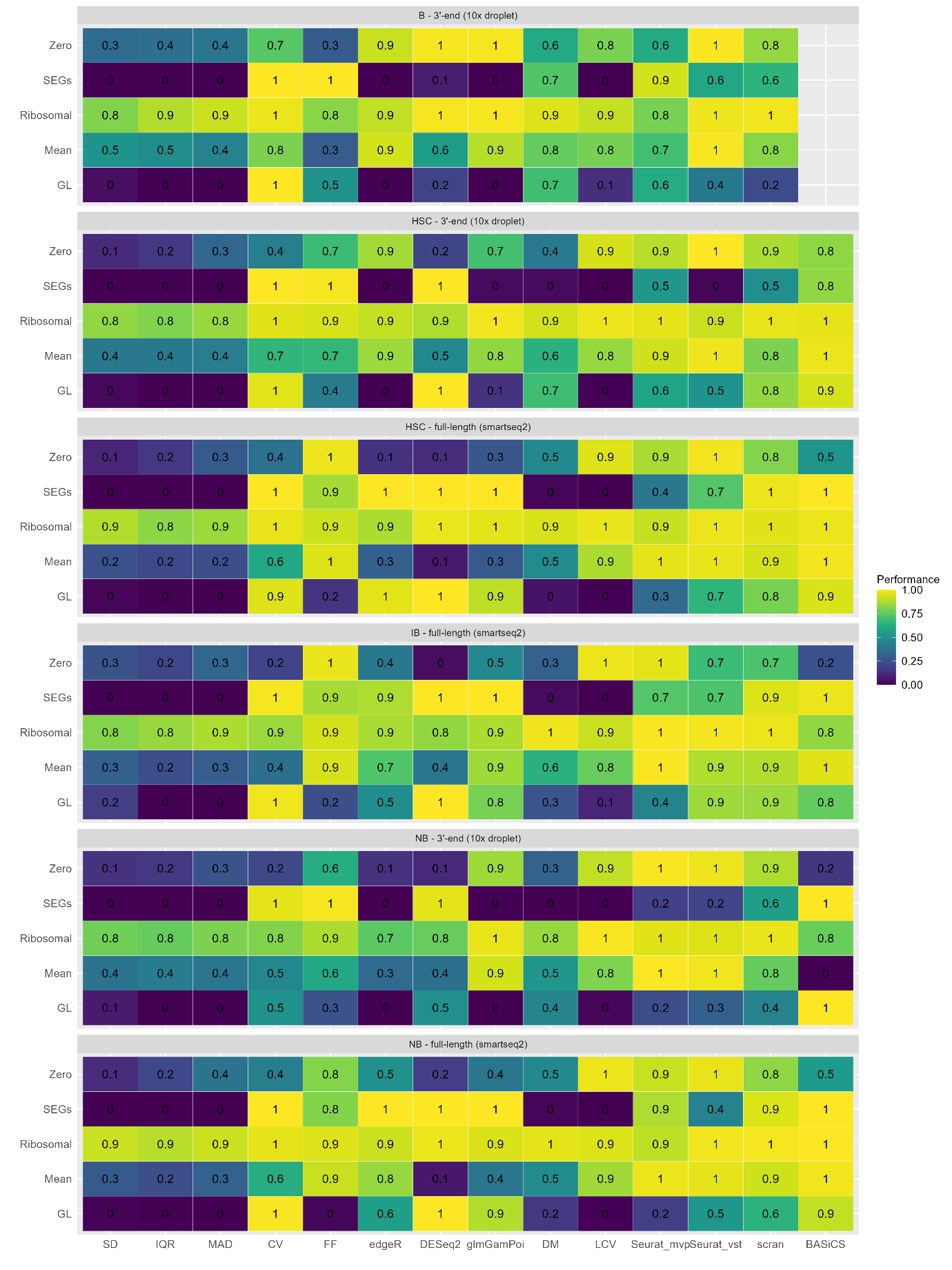


Fig. S2. Heatmaps of metrics performance on three cell types with two sequencing platforms. Each role represented five main criteria which included proportions of zero per gene (zero), mean expression (mean), ribosomal genes (ribosomal), stably expressed genes (SEGs) and gene length (GL). Metric was highlighted by different categories. Data used for each evaluation heatmap was labelled on each panel, where the first one represented 10k human B cell data and the rest were from TMS datasets. BASiCS was not applied to large human B cells as lacking batch information and computational complexity.

**
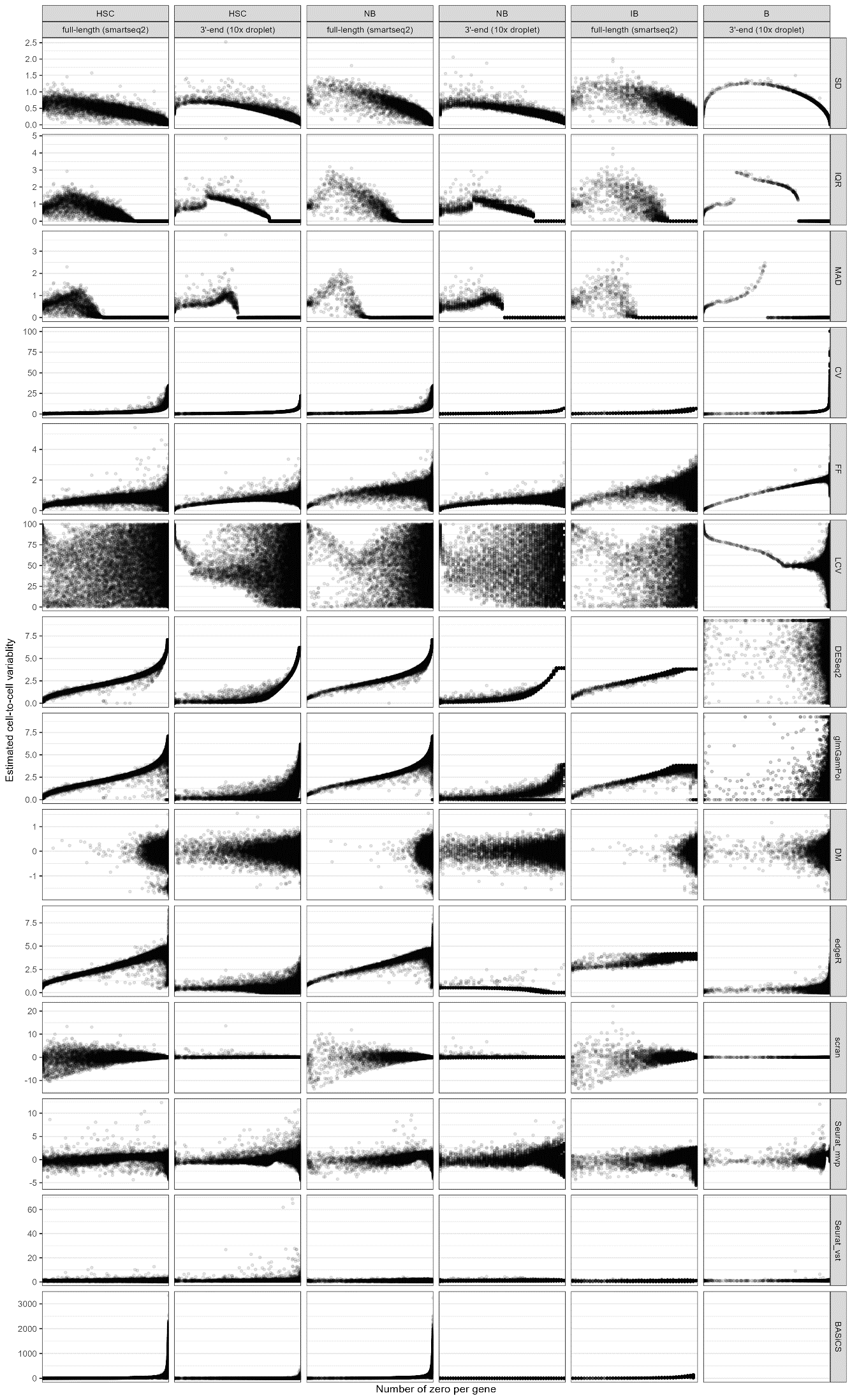
a**

**
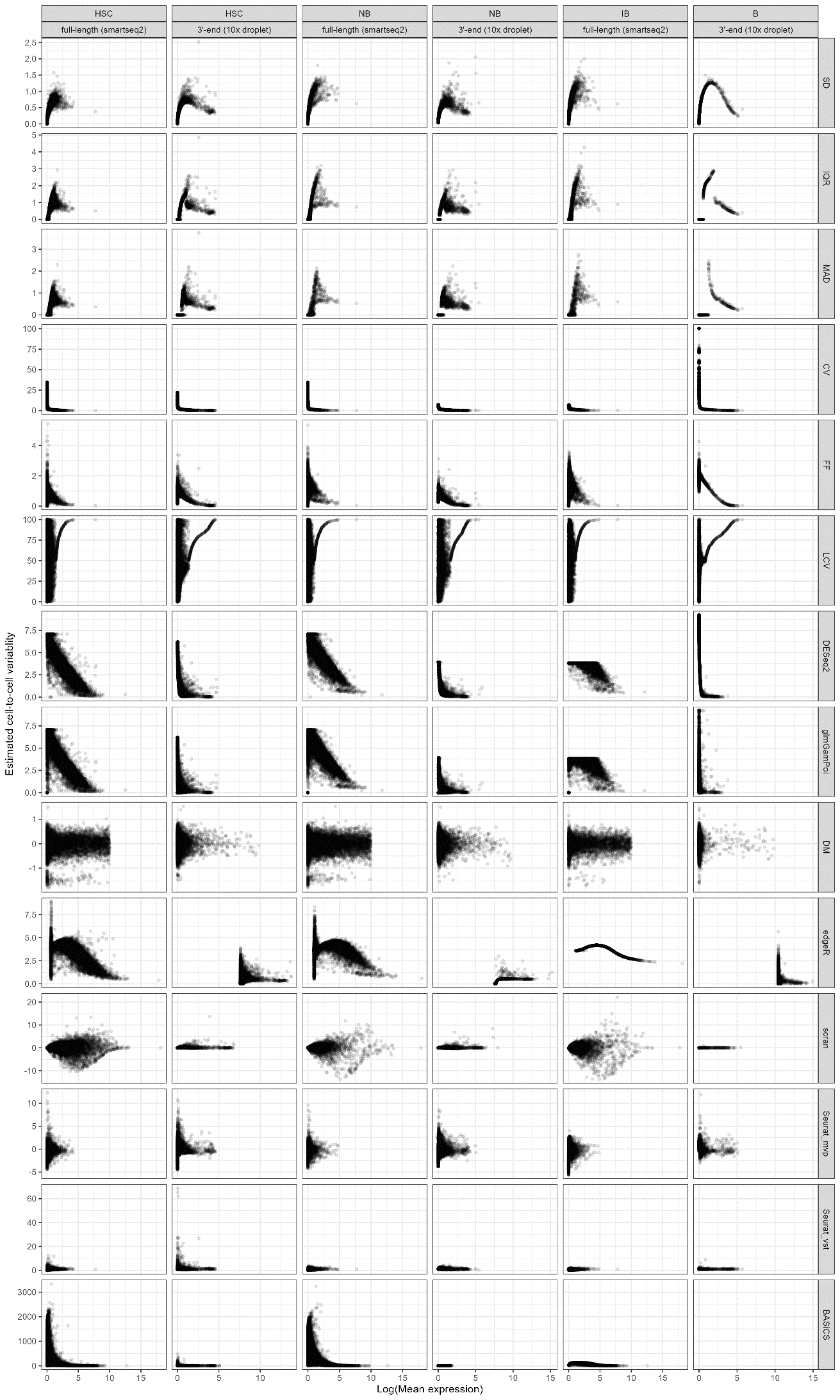
b**

**
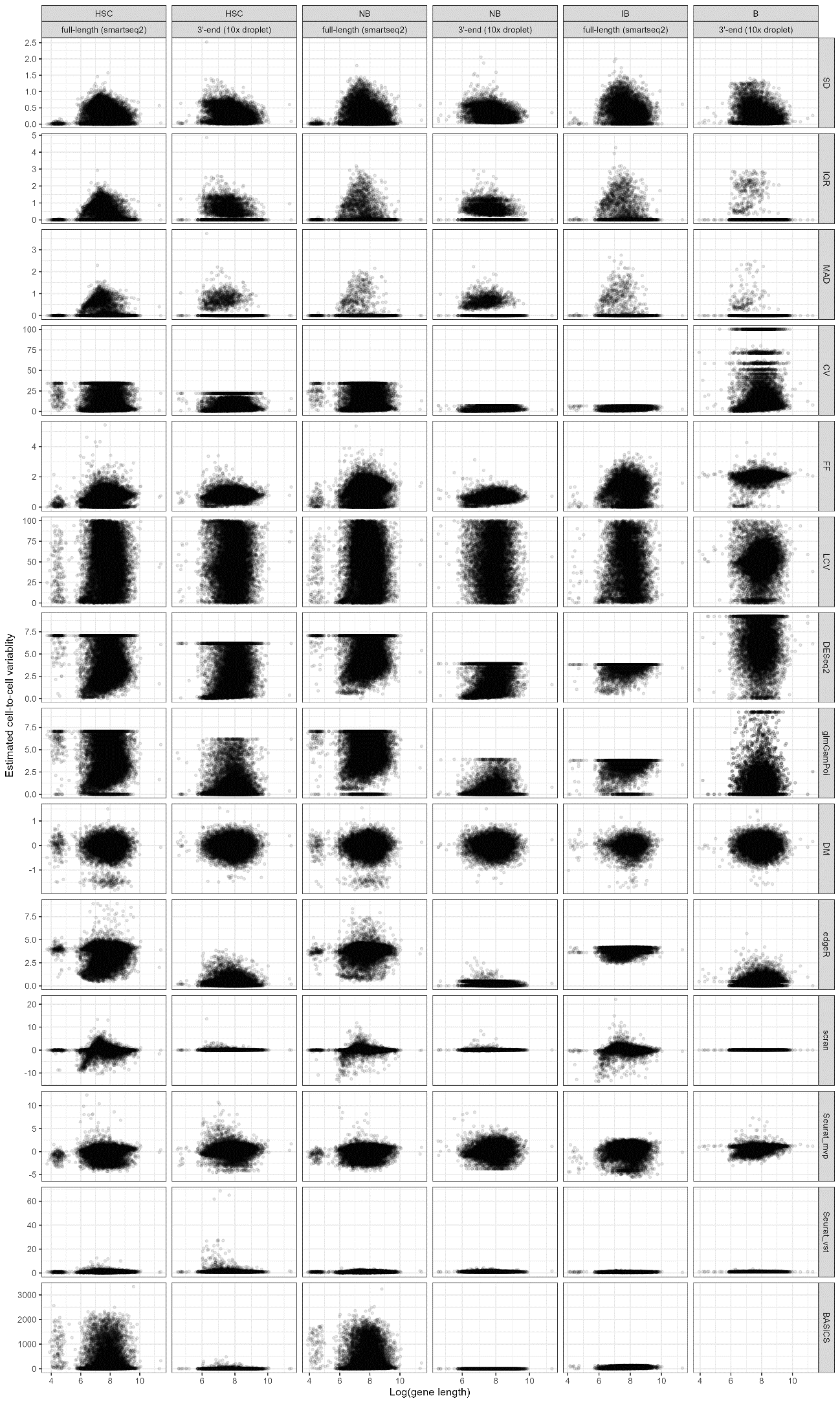
**c

**
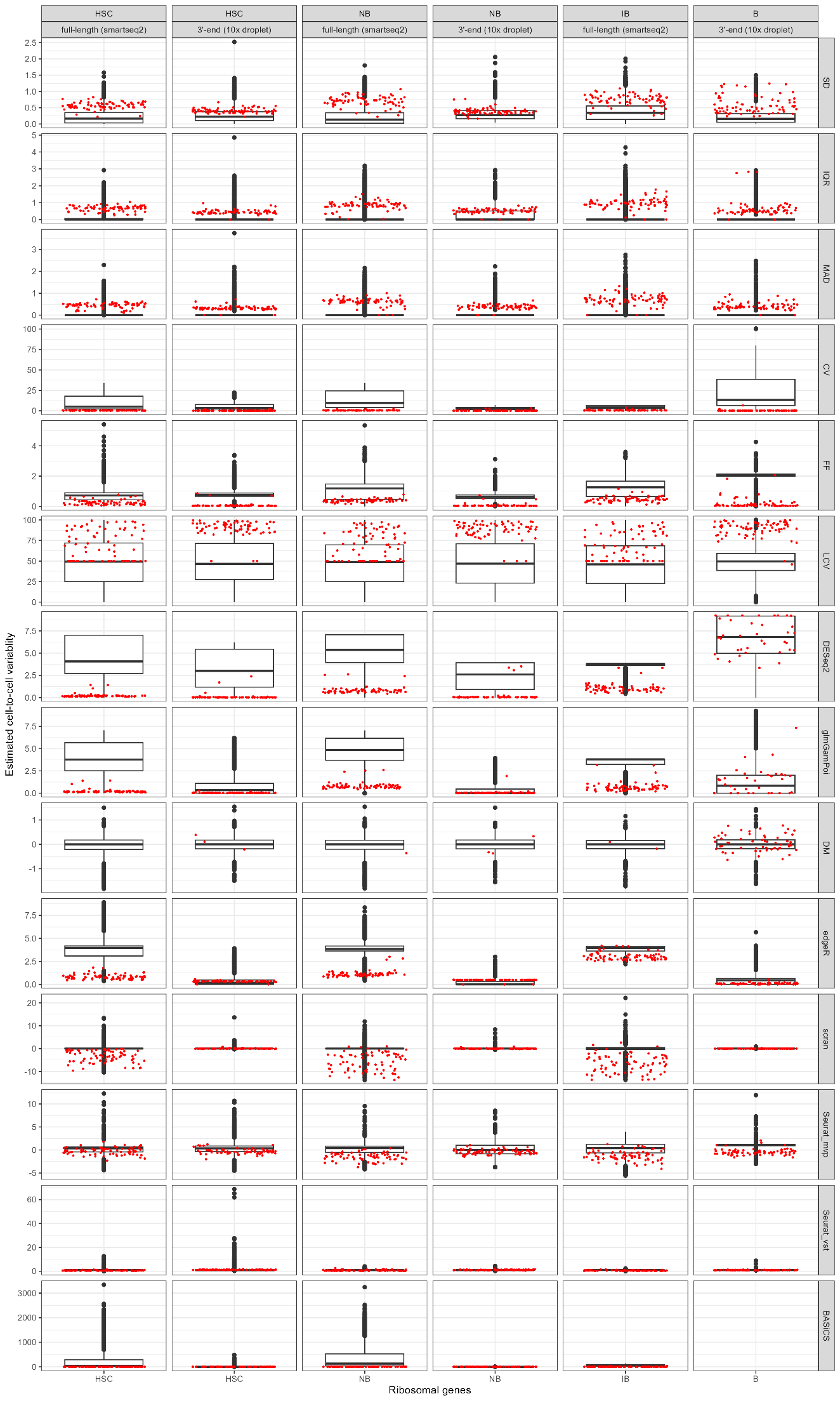
d**

**
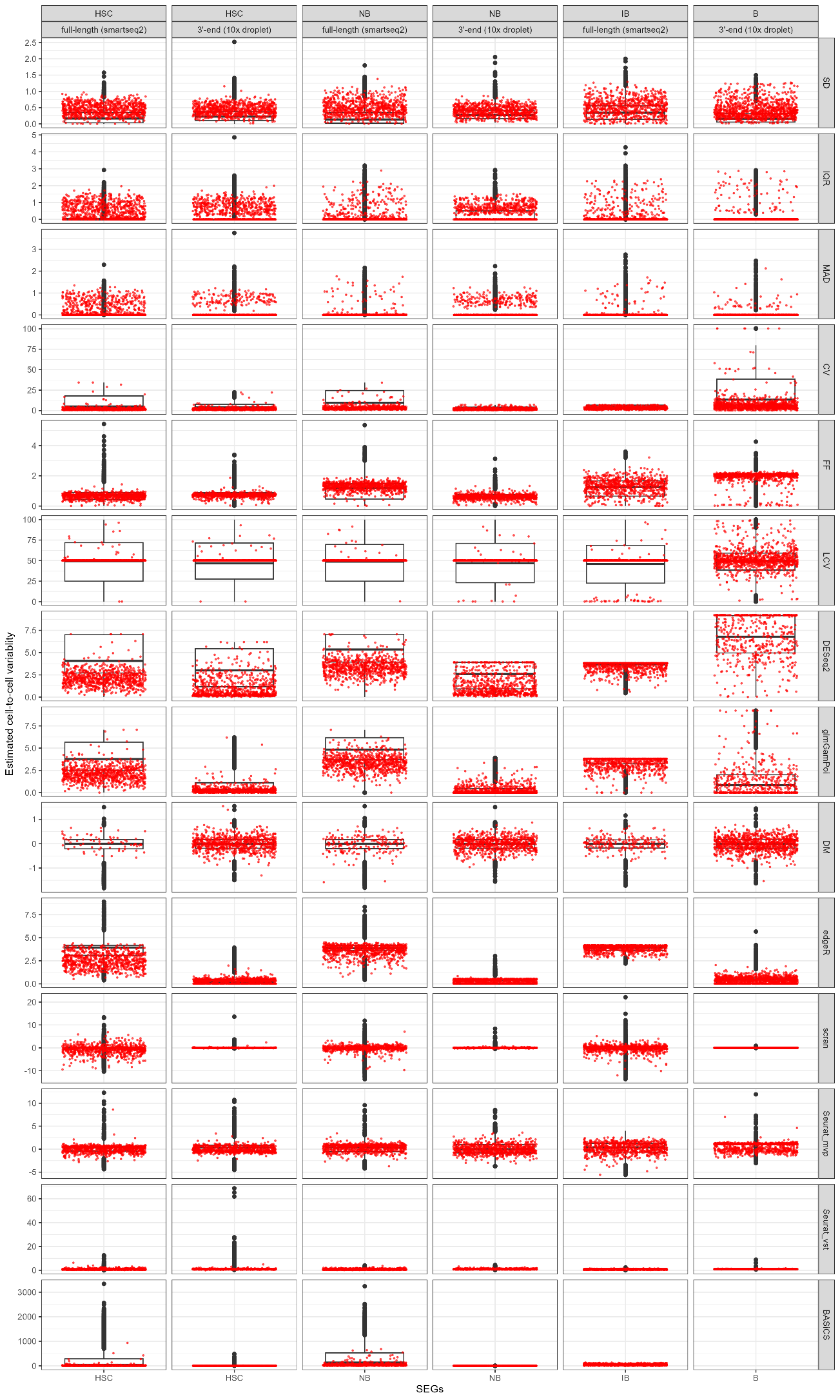
e**

Fig. S3: Plots for each metric between the estimated cell-to-cell variability and each aspect of data characteristics. Each dot in the plot represented a gene. Each row represented one dataset that had been tested. a) Dot plots for estimated cell-to-cell variability against the number of zero per gene across all cells. b) Dot plots for estimated cell-to-cell variability against the log mean expression. c) Dot plots for estimated cell-to-cell variability against available gene length. d) Boxplots for each measured cell-to-cell variability with ribosomal genes coloured in red. e) Boxplots for each measured cell-to-cell variability with stably expressed genes coloured in red. Note, BASiCS was not applied on human B cells due to the large sample size resulting in computational burden.


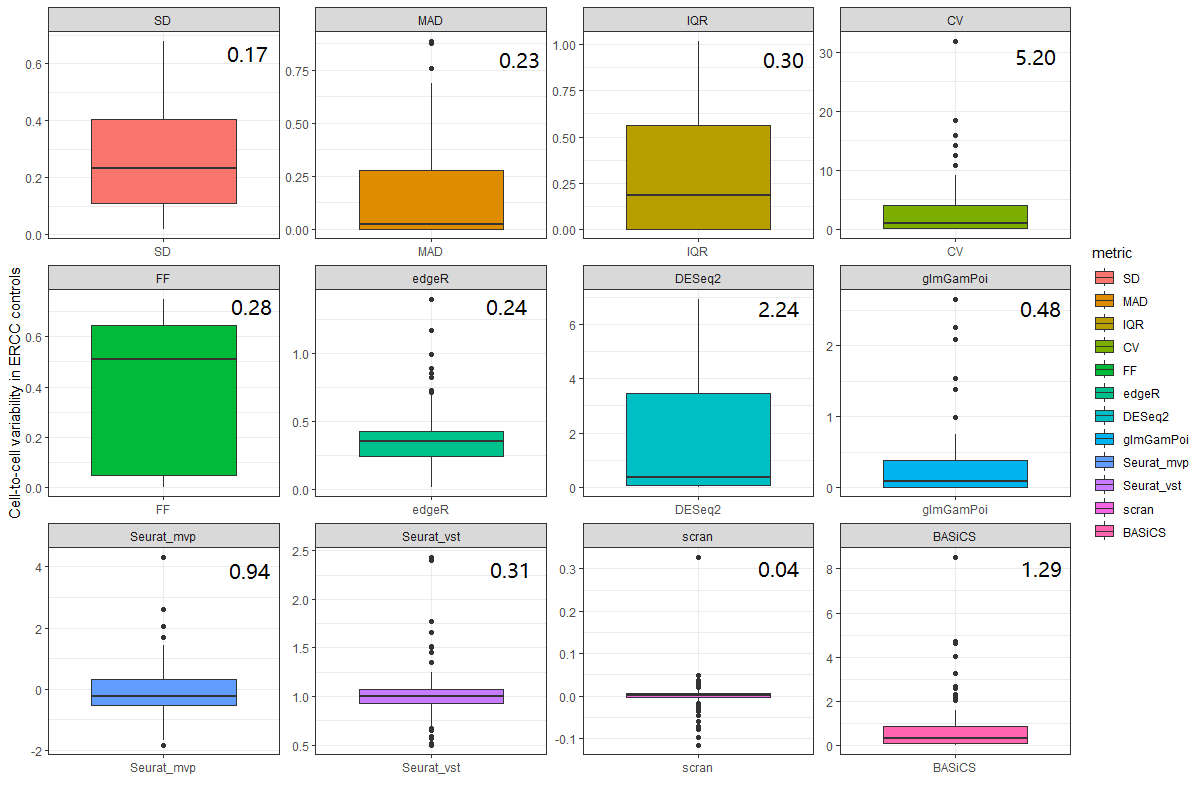


Fig. S4: Metrics measured on the spike-in ERCC control dataset that only contained technical variability as shown in the boxplots. The measurement standard deviation was calculated and listed for each metric in the figure. The lower dispersion value illustrated the capability of measuring biological variability rather than technical variability by a metric. DM and LCV were not included as required gene length and sufficient gene number for smoothing, respectively.


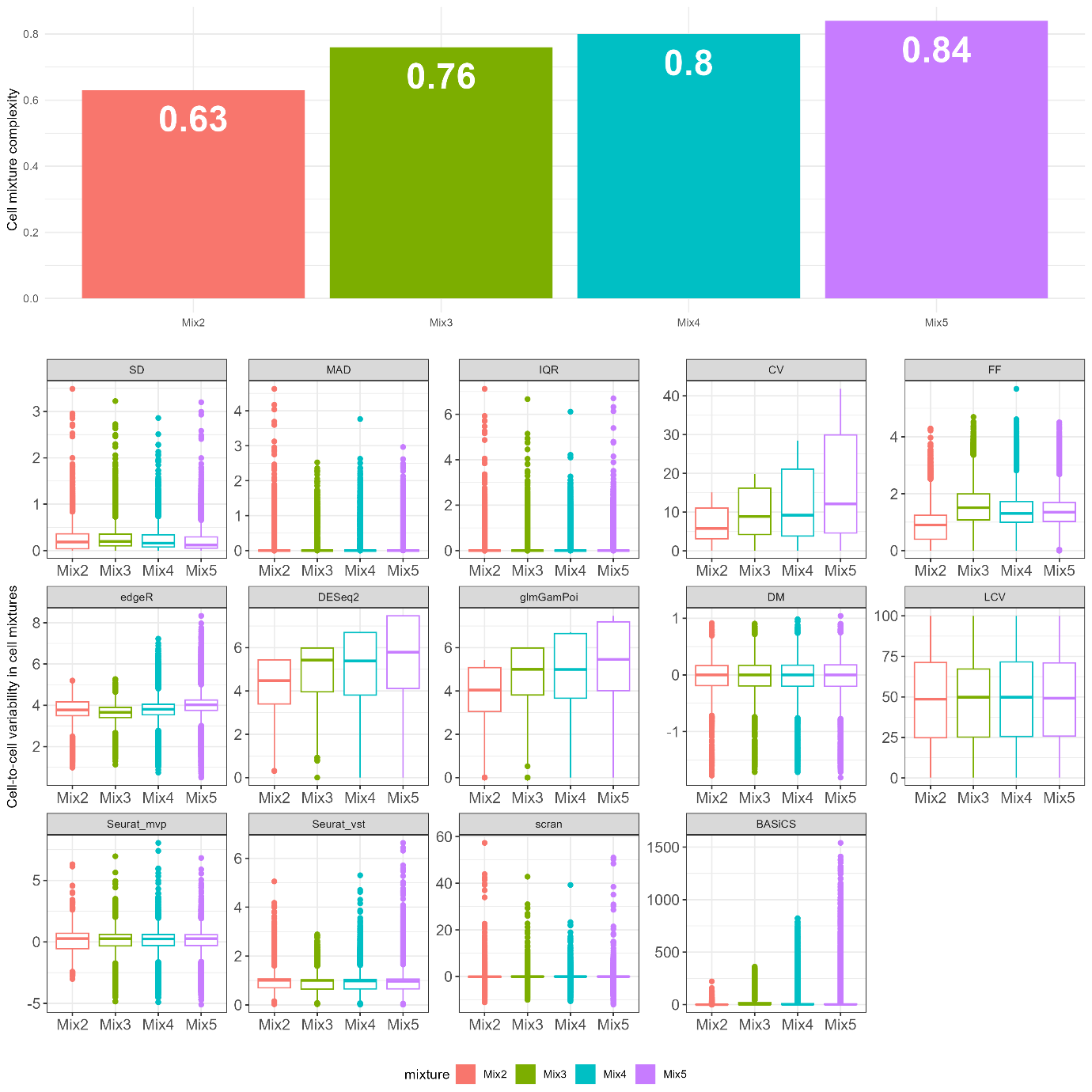


Fig. S5. Metric performance in cell mixtures with different levels of data complexity. Mix2-5 represents the number of cell types in bone marrow tissues that with at least 100 cells. a) The data complexity in each mixture was listed in the barplot. b) Boxplots showed the cell-to-cell variability in each mixture for 14 metrics.
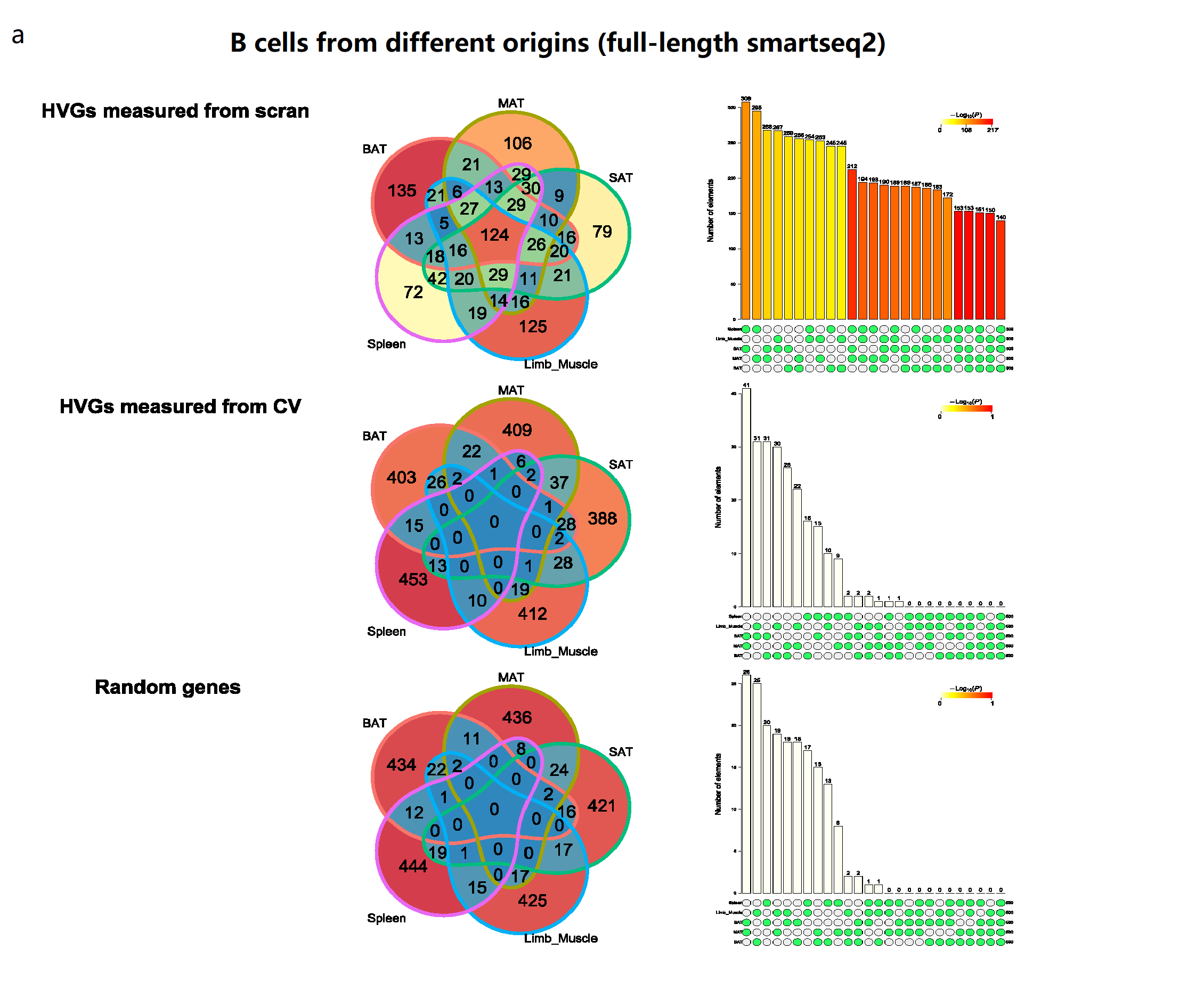


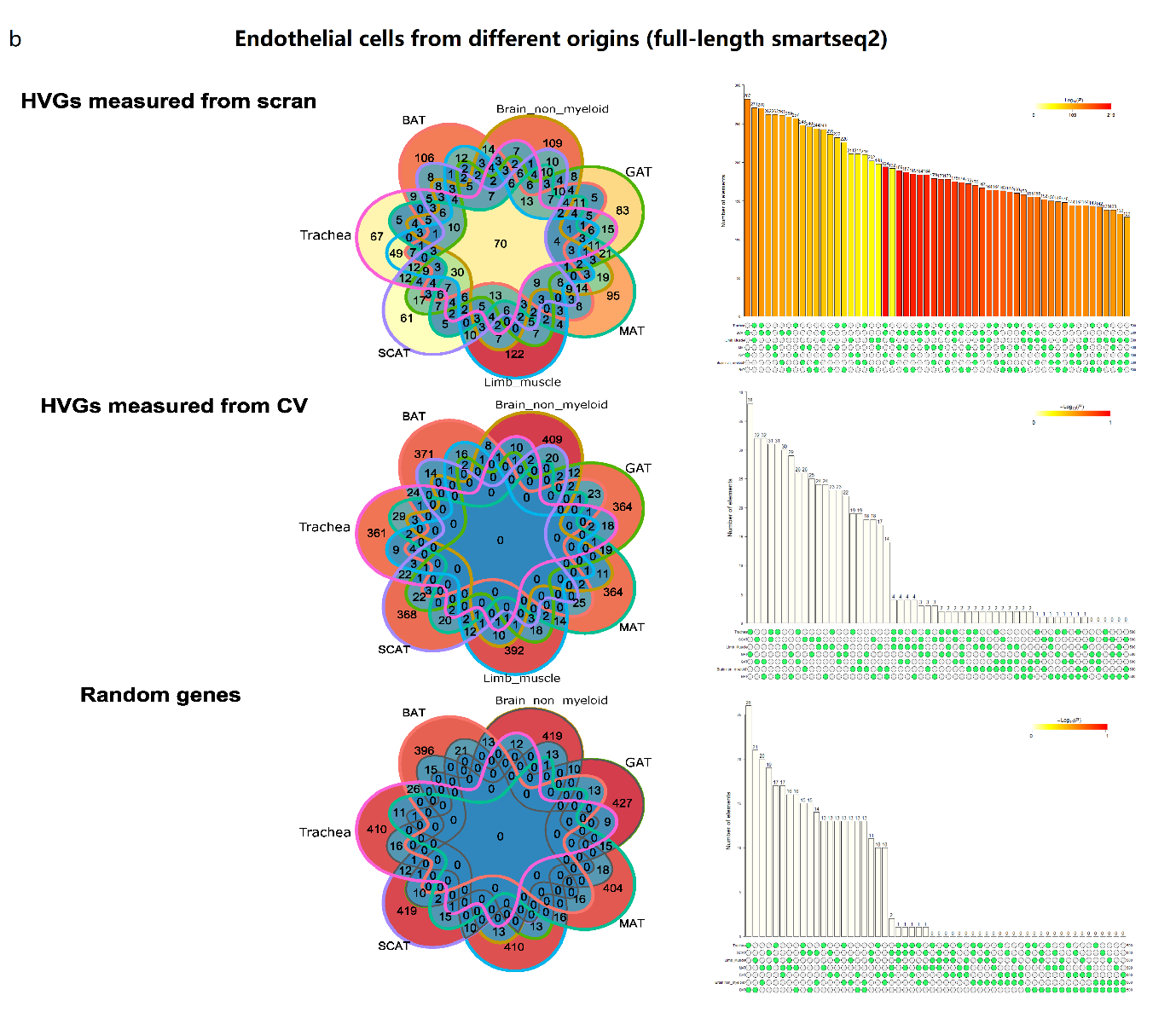


Fig. S6: Venn diagrams showed the overlapped percentage of highly variable genes (HVGs) for B cell (a) and endothelial cells (b) measured by scran across multiple tissues (BAT: brown adipose tissue; MAT: mesenteric adipose tissue; SCAT: subcutaneous adipose tissue; GAT: gonadal adipose tissue;) and barplot showed the overlap of every listed with the colour indicated p-values. By further comparing with random sampling genes, the overlap of HVGs captured with random gene lists showed high similarities with CV but not scran.


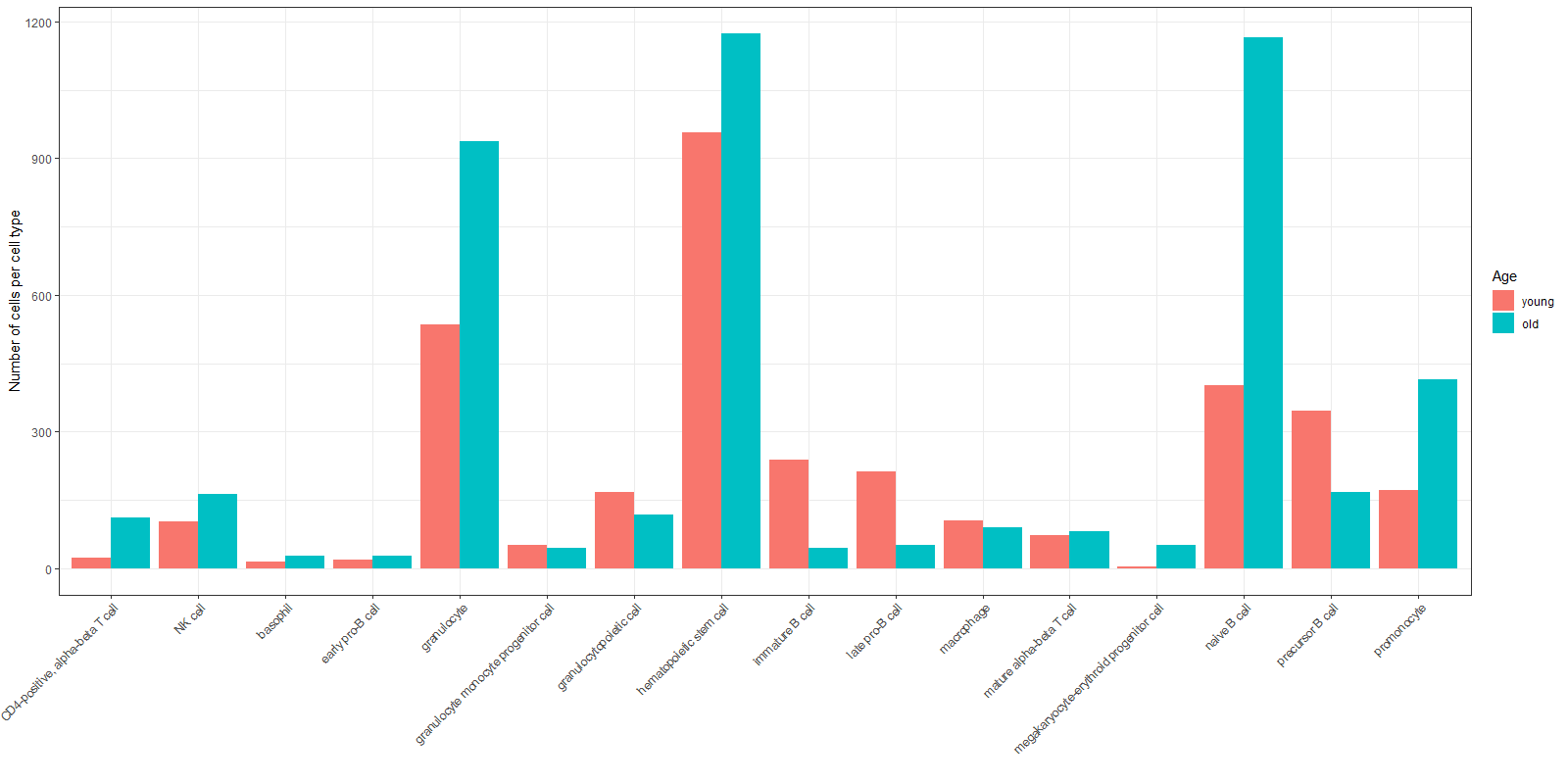


Fig. S7: Barplot illustrated the number of cells in the marrow tissue that have been sequenced by facs-smartseq2 for both young and old groups in TMS. To investigate the B cell lineages differentiation and aging, only related cell types with more than 100 cells in either age group were included, which were HSC, late progenitor-B, precursor B, immature B, naïve B cells, promonocytes and granulocytes. As we aimed to investigate variability changes under differentiation trajectory, promonocytes and granulocytes were excluded from the analysis.

Fig. S8: Volinplot of gene expression variability for five cell types between young and old groups from Tabula-Muris-Senis data. Out of five cell types, only precursor B
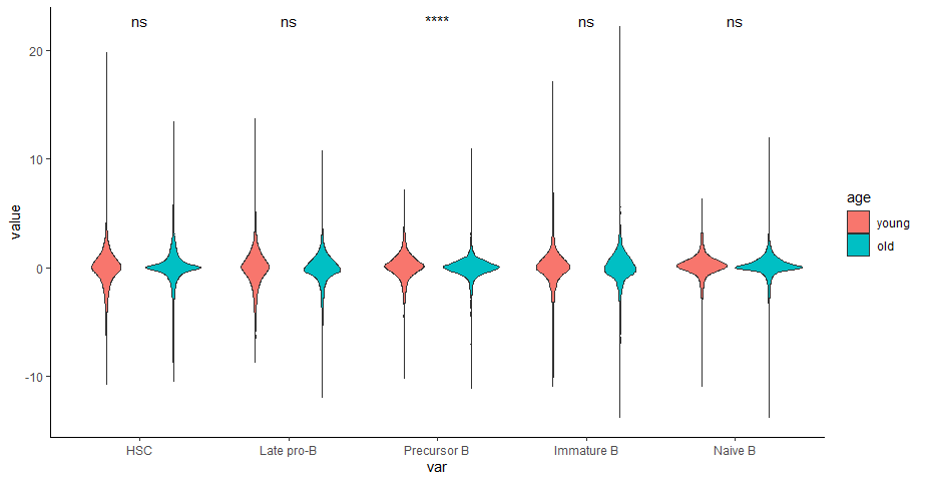
 cells showed a significant difference between young and old groups by performing the Wilcoxon ranked sum test. ns represented no significance and **** represented p.value < 0.0001.


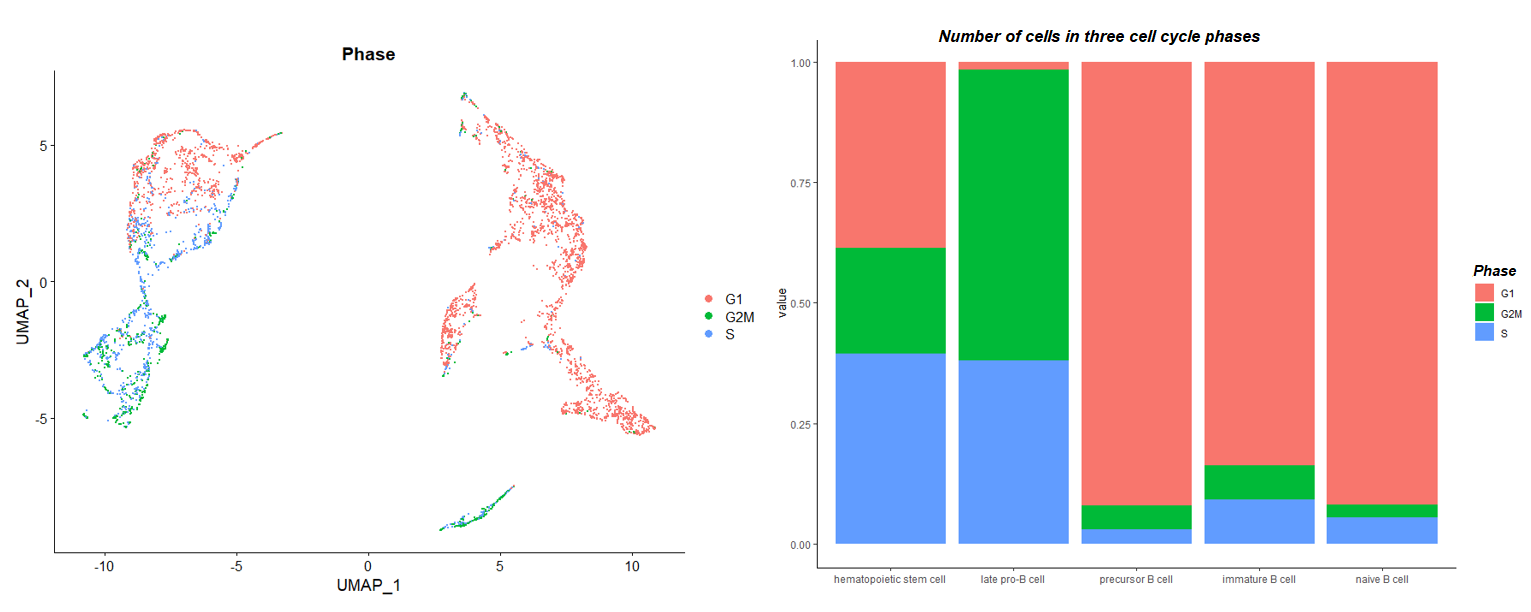


Fig. S9: The cell cycle stages of HSC, late pro-B cells, precursor B cells, immature B cells and naïve B cells. Stages were assigned to each cell and coloured in the UMAP. Barplot showed the corresponding proportions of cells in the G1, G2M or S phase.


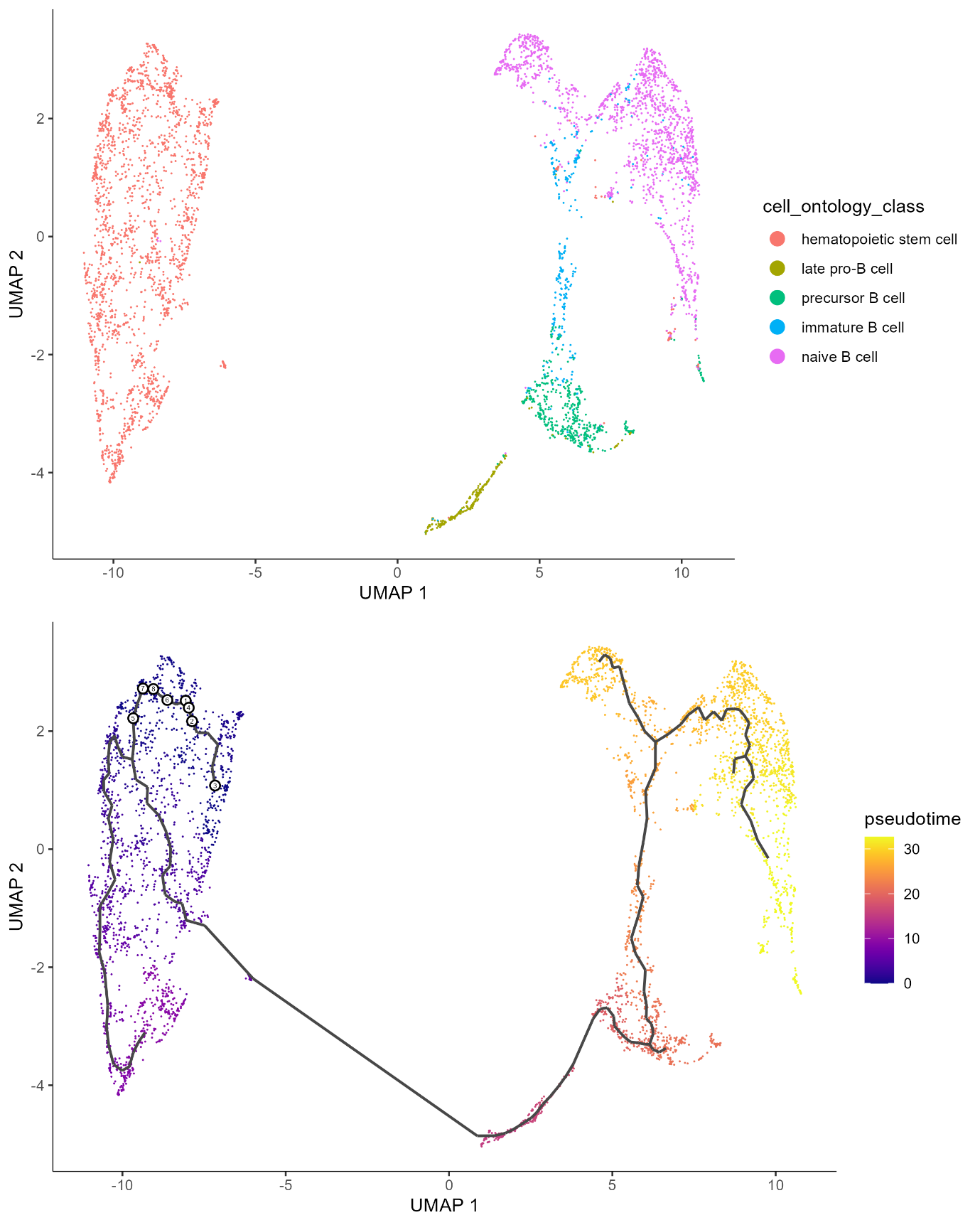


Fig. S10: UMAP illustrated the pseudotime inference for each cell type after removing the age effect. The pseudotime trajectory matched the biological B cell differentiation, however, naïve B cells showed outspread pseudotime inference.


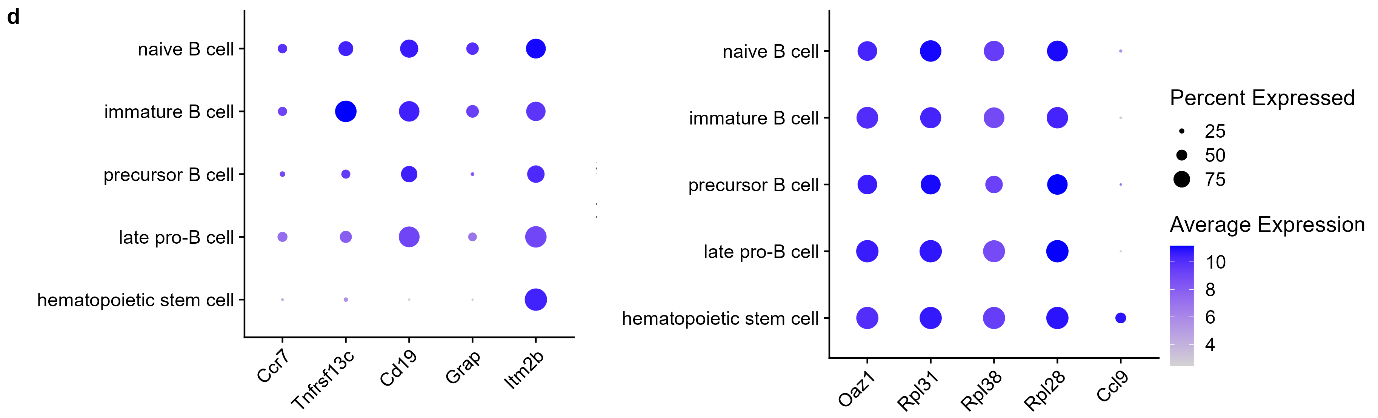

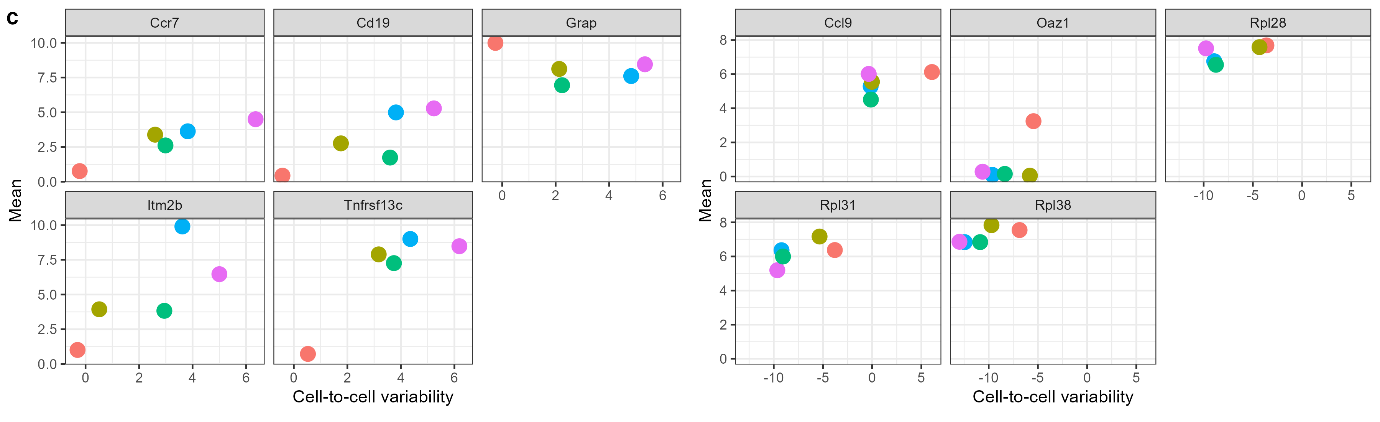

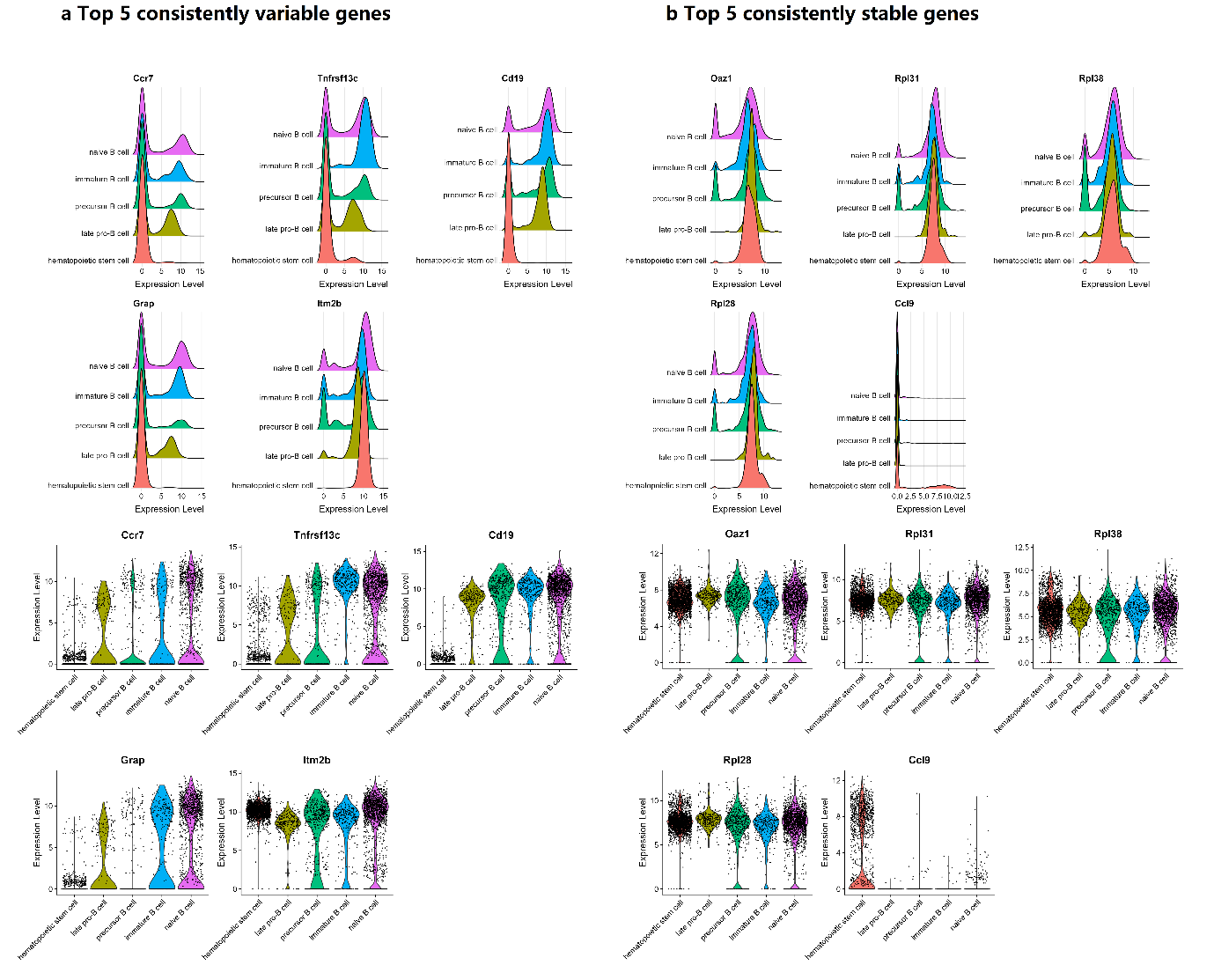


Fig. S11: Details of the top 5 consistently variable genes and consistently stable genes along the B lymphocytes differentiation process. Ridgeplot of expression and violin plot of the average gene expression in each cell type for a) consistently variable genes, b) consistently stable genes. c) Scatter plot between the cell-to-cell variability and mean expression for each gene along the differentiation process. d) Dotplot demonstrated the percentage of expression in each cell type for these marker genes, coloured by mean gene expression level.


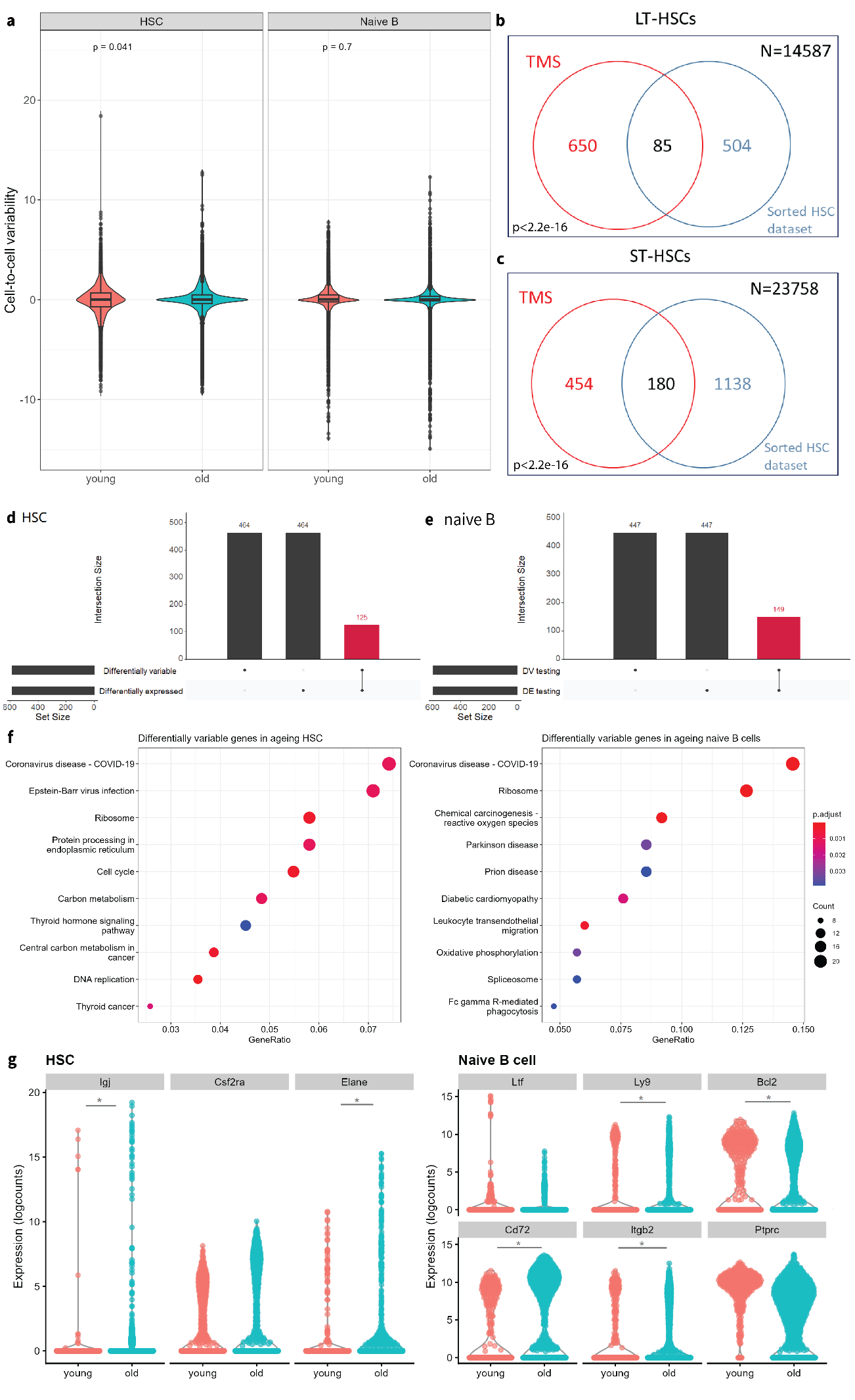


Fig. S12: Cell-to-cell variability alterations in HSC and B lymphopoiesis in aging. a) Overall distribution of measured variability in HSC and NB between young and old groups. P-values were calculated by the Wilcoxon test for each cell type. Venn diagrams of DV genes from TMS HSC and an external sorted b) LT-HSCs and c) ST-HSCs [1]. The upset plot demonstrated the overlaps between DV genes and DE genes during aging in d) HSC and e) naïve B cells. f) Top 10 significant pathways from KEGG databased based on significant DV genes from HSC (left) and naïve B cell (right), ordered by p-value. g) Violin plot highlighted the gene expression level of the targets of *Sfpi1* between young and old groups for HSC and naïve B cells. * - adjusted p-values <0.01.

a Full-length smartseq2 technology


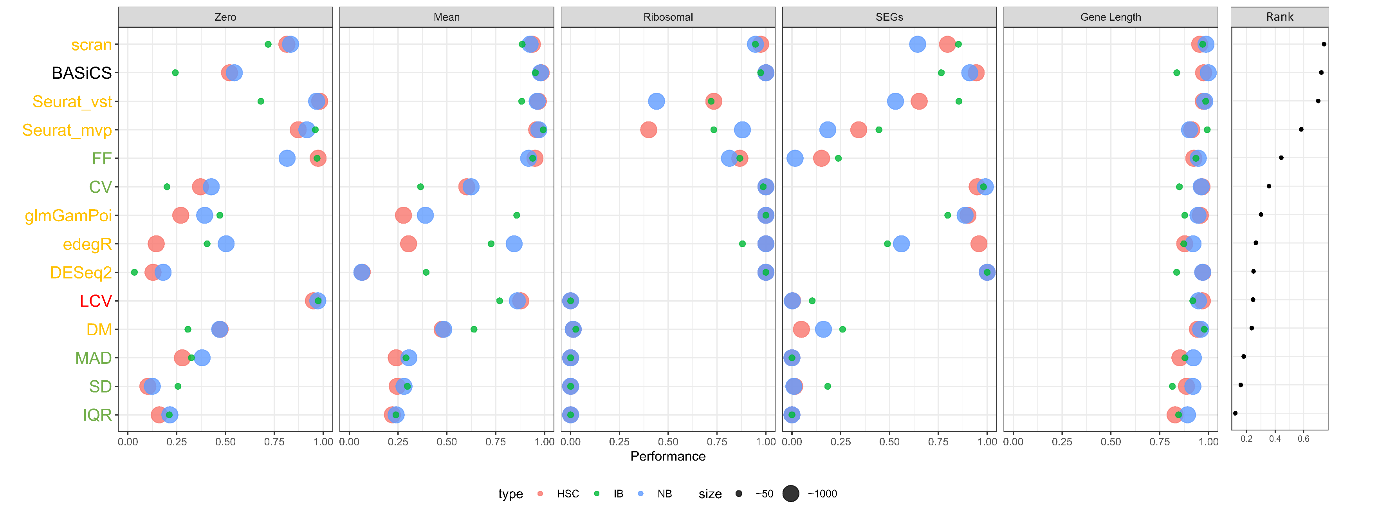


b 3’-end 10X droplet technology


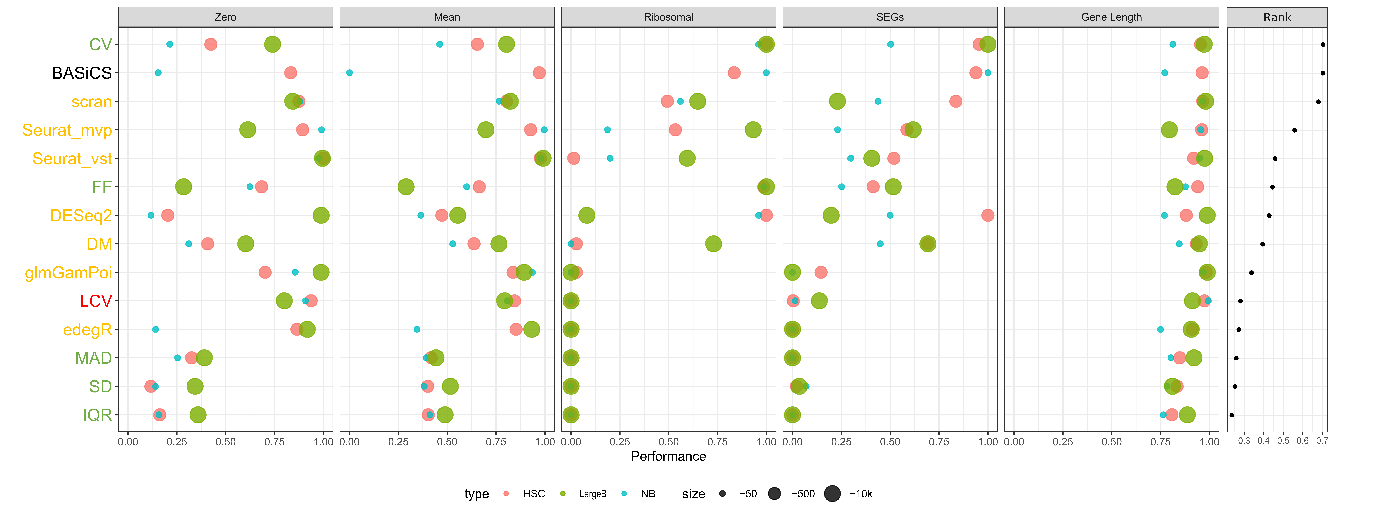


Fig. S13. Metric performance evaluated per sequencing platform. Complementary to the overall evaluation performance for each metric, the joint dotplot showed the metric performance in a) full-length smartseq2 technology and b.) 3’-end 10X droplet technology with the metric ordered by the ranks and coloured by categories.
