# Supplementary material for: Measuring cell-to-cell expression variability in single-cell RNA-sequencing data: a comparative analysis and applications to B cell aging
Source: Genome Biol. 2023 Oct 20;24:238. doi: 10.1186/s13059-023-03036-2 (PMC10588274; doi:10.1186/s13059-023-03036-2)
Supplement: Supplementary file 2 — Additional file 2. Review history. [file 13059_2023_3036_MOESM2_ESM.docx]

Review History

**First round of review**

**Reviewer 1**

**Were you able to assess all statistics in the manuscript, including the appropriateness of statistical tests used?** Yes, and I have assessed the statistics in my report.

**Comments to author:**

In this manuscript, Zheng et al. systematically compared 14 variability metrics for evaluating cell-to-cell gene expression variability from single-cell RNA-seq data. The authors then use the metric with the best performance (scan) to investigate the changes in cell-to-cell variability associated with aging using scRNA-seq datasets from HSCs and B lymphocytes. My major concerns include the quality of the analysis and the lack of experimental validations. In addition, there are several comments that should be addressed before the publication of the manuscript:

1. To investigate the impact of sample size on the performance of metrics, the authors should use sub-sampled data (with different numbers of cells) from the dataset of the same cell type.

2. The authors should test their metrics on positive controls with high biological variations (e.g., datasets with multiple cell types included vs. a single cell type).

3. Cells in the UMAP (supplementary figure 8) are separated by clusters and not organized into trajectories.

4. For genes that show high or low variability along B cell differentiation, are their stability associated with their expression level in different B cell state?

5. The authors detected slightly decreased variability of gene expression in the aged HSC. Is this in contrast with the previous report that aging is associated with increased transcriptome noise?

6. How does the target TF (Sfpil) expression level change during aging for HSC and naive B cells? Is the expression changes associated with the variability of this gene?

7. The text fonts in many figures are too small to see (e.g., Supplementary Figures 3, 5, and 7). These should be improved.

**Reviewer 2**

**Were you able to assess all statistics in the manuscript, including the appropriateness of statistical tests used?** Yes, and I have assessed the statistics in my report.

**Comments to author:**

Zheng et al. have described a benchmarking study in which they have compared 14 metrics for assessing cell-to-cell expression variability in single-cell RNA-sequencing data. After benchmarking all these methods, they have picked the top-performing method (scran) to assess cell-to-cell expression variability during HSC-to-B-cell differentiation and aging in B-cells and HSCs from young vs old mice.

One of the unique properties that can be assessed in scRNA-seq data as opposed to bulk RNA-seq, is the cell-to-cell expression variability. Measuring this could reveal biology that has previously been hidden. However, a systematic comparison of cell-cell expression variability metrics in scRNA-seq data was something that was not conducted before. This has made it difficult so far to interpret studies using different metrics for assessing cell-cell expression variability, and therefore, I believe this study is a valuable addition for the field.

In general, I enjoyed reading the study, and most of the reasoning has been clearly explained. However, there is a few points that could further strengthen the study or enhance readability:

1. The labeling of Figures can be a bit more consistent across figures. For example, Fig. 1 says FACS vs droplet, Fig. 3 10xdroplet vs smartseq2, Fig. 6 3'-end vs full-length, etc. For accessibility for a broader audience, I suggest to use the description: full-length (smartseq2) vs 3'-end (10x droplet).

2. Fig. 1B is a bit difficult for me to interpret. I don't understand what is on the x- and y-axis or how I should interpret the lines etc. I can read the conclusions, but by looking at the figures themselves I couldn't clearly see the conclusions that were drawn (but that might just be me not understanding the actual figures).

3. Fig. 2B - DM method: what does a negative cell-to-cell variability value implicate here? It's not like you are comparing two conditions, so it's a bit unclear what this means?

4. Fig. 3: Based on this Figure, it seems that the performance for Smartseq2 vs 10X Genomics data differs quite a lot for certain methods. And therefore, I wonder whether a separate ranking should be visualized for these 2 methods instead of averaging the ranking (it's not common that both approaches are used in the same study). In general, the largest sample size seems top performing in each of the tested classes of parameters - however, scran -10x genomics - largest sample size is performing worst. How do you explain this (especially since you're suggestion is to use scran as top performing approach, it would be good to understand when it may deviate)?

5. The labeling of the Figure axes is sometimes absent (but can be extracted from the title of the figure). However, Fig. 4C y-axis for example is unclear.

6. Figure 6 gives a nice overview of what has been done in the study, but having it as the last figure is strange for a study overview (then making it Figure 1 would make more sense). It would be very valuable to make this figure a summarizing figure instead, so including advice for the reader which approach to use depending on the dataset characteristics (10X droplet vs FACS smartseq2, dataset size, dropout rate, etc).

7. In the following text: 'Overall, there was a slight decrease in variability observed in old HSC compared to young HSC (Wilcoxon test, marginal P-value = 0.04, Supplementary Figure 10a) and no significant effect in naïve B cells (Wilcoxon test, marginal P-value = 0.7, Supplementary Figure 10a).' What is meant by 'marginal p-value'?

8. 'To increase the power for calculating DV genes, we first pre-filtered the genes based on the proportions of zeros.' Please specify in more detail which cut-off was chosen.

9. Pathway overrepresentation analysis: unclear what background gene set has been used to compare against. Please specify in the methods section.

10. Would be interesting to learn how the DV results compare to the DE results for the same comparison (e.g. aging analysis). Do you identify new sets of genes that weren't detected by one or the other?

11. How should I interpret the DV results in general? Looking at the results, it seems that many of the genes are associated with subcelltype composition: 'Three cell differential markers, Ccr7, Tnfrsf13c, Cd19, showed the greatest variability

changes along the differentiation process'. So is it truely biological variability that increases, or is the accuracy of cell type annotation also disturbing the analyses?

12. Suppl Table 2: Should the D-statistic be as small as possible when comparing same cell type across scRNA-seq platforms, and as high as possible when comparing different cell types across the same scRNA-seq platform? In other words, are you kind of comparing the influence of technical vs biological variability on the precision of the cell variability metric? Maybe you can aid the reader here by making more clear which comparisons you would like to have an as low as possible vs an as high as possible D-statistic (color-code where green is best/red is worst)?

13. Suppl. Fig.1: better to align the last block (largeB droplet) with the top ones, that eases comparison across the different cell types.

14. For some figures, e.g. Fig. S4, S5, S6, etc., it is not directly clear just looking at the figure which data has been used as input (especially whether it's smartseq2 or 10x data would be good to know as the performance of some metrics seem to be better for one than the other). Just adding this information in the legend (when it's not already in the figure itself) would be helpful.

**Authors Response**

**Point-by-point responses to the reviewers’ comments:**

Responses to Reviewers’ Comments

We thank both reviewers for their constructive feedback and taking the time to undertake this review. We have included both a clean and tracked changes version of the revised manuscript. All page and line references here in this rebuttal refer to the clean version.

**Reviewer #1**

In this manuscript, Zheng et al. systematically compared 14 variability metrics for evaluating cell-to-cell gene expression variability from single-cell RNA-seq data. The authors then use the metric with the best performance (scan) to investigate the changes in cell-to-cell variability associated with aging using scRNA-seq datasets from HSCs and B lymphocytes. My major concerns include the quality of the analysis and the lack of experimental validations. In addition, there are several comments that should be addressed before the publication of the manuscript:

We thank the reviewer for their assessment and constructive feedback that have strengthened this manuscript. The specific questions and suggestions raised by the reviewer have been addressed as comprehensively as possible. Regarding the first major concern, we have implemented additional analyses at the request of the reviewer and as a result, this has greatly improved the quality of the overall analysis in the manuscript. Regarding the second major concern, we have validated the set of differentially variable genes using an external dataset and this has been a significant extension to the manuscript. Specifically, we used an external scRNA-seq dataset that profiled long-term and short-term HSCs in young and old mice and detected a significant overlap with the differentially variable genes from the old versus young HSCs in the Tabula Muris Senis dataset. This confirms the robustness of the analysis and provides an appropriate validation of these results.

We acknowledge that the reviewer expressed their concern in the context of experimental validation and that we have instead taken an approach that leverages an externally derived dataset. This decision was based on the consideration of several factors. First, as a small computational lab, it would take a significant amount of time, costs, and resources including the recruitment of an appropriate collaborator who would be able to provide access to the experimental assays and expertise in using them that we currently do not possess and in mouse HSCs. While this is not impossible, it certainly increases the unpredictability of timeline management for completing this manuscript revision that we felt we could not reasonably justify. Second, the manuscript is fundamentally focused on a computational study. We identify the best-performing metric based on an extensively designed benchmarking study for estimating gene expression variability from scRNA-seq data and apply this metric to understand how gene expression variability contributes to the cell type-specific regulation of a complex phenotype which in this case is ageing. While experimental validation of the results, especially for the differentially variable genes, would surely add some value to confirming the identity of the proposed targets, the emphasis and impact of this manuscript lies in the computational approach which have been strengthened by the validation using the external dataset. We have revised the Abstract and Conclusion to further emphasise this point of the manuscript (see page 1, line 19, and page 18, line 552, respectively). Third, the provision of publicly accessible scRNA-seq datasets has become such a commonplace practice now that identifying an appropriate external dataset for validation is a faster and more efficient alternative to generating new datasets. In response to this reviewer’s request, we searched for publicly available datasets that might be appropriate based on design (sufficient sample size, comparable variables) and relevance (same organism, cell type, platform), and were able to find a suitable candidate even though FACs-sorted large RNA-seq datasets for the bone marrow have not been extensively investigated. The fact that our results had a significant overlap with those obtained from the external dataset reinforces the robustness of this result and moreover provides appropriate validation. Fourth, as long-term readers of Genome Biology, we are aware that there have been other papers recently published that have used external datasets as a validation strategy instead of an experimental system. Specifically, we were inspired by these papers based on how their studies were structured with respect to validation.

Li et al. (2023). <https://genomebiology.biomedcentral.com/articles/10.1186/s13059-023-02897-x>

Karollus et al. (2023). <https://genomebiology.biomedcentral.com/articles/10.1186/s13059-023-02899-9>

Bhuva et al. (2019). <https://genomebiology.biomedcentral.com/articles/10.1186/s13059-019-1851-8>

We hope that our rationale is comprehensive enough for the reviewer to consider the validation approach that we have adopted to be a reasonable approach.

We have updated the manuscript with an additional paragraph that reflects this validation, please see page 14, line 414:

“We conducted a validation of the DV genes in aged HSCs from the TMS data by comparing them to an external dataset which contains sorted long-term HSCs (LT-HSCs) and short-term HSCs (ST-HSCs) from young and old mice (see Methods) [32]. The significant overlaps between the DV genes from the TMS data and the two types of HSCs indicate the robustness of the markers detected across the TMS datasets (hypergeometric test, P-value < 0.05; Supplementary figure 12b and12c) and provides a validation of our result. Interestingly, we identified a higher number of DV genes in ageing ST-HSCs with respect to the ageing LT-HSCs which supports the implication of prompt differentiation function in ST-HSCs but quiescent states in LT-HSCs [33]. Additionally, we examined whether the DV genes identified in TMS showed differential expression (DE) in terms of mean expression between the young and old groups. While the number of DE genes was substantially higher than the number of DV genes, we found at least 20% unique DV genes with no significant mean differences during ageing in HSCs and naïve B cells (Supplementary figure 12cd and 12e).”

1. To investigate the impact of sample size on the performance of metrics, the authors should use sub-sampled data (with different numbers of cells) from the dataset of the same cell type.

This is an excellent suggestion because it provides an opportunity to extend the existing analyses to test the metric performance based on sample size using fixed numbers of cells. We subsampled HSCs from the smartseq2 dataset into 10%, 20%, 50%, 80% and 90% of the total cell populations and repeated each procedure 5 times. To determine performance, we compared these results back to the variability observed in the full data. Please see page 7, line 198 where an extra paragraph was added to describe these results under *Investigating the impact of sample size on metric performance* section, also see Supplementary figure 1.

“To further investigate the impact of sample sizes on the same cell type, we subsampled HSCs sequenced by two platforms from 10% to 90% of the total cell number and compared them with respect to full data. We found the distance between sub-sampled and full data decreased with the increasing number of sample sizes and reached a steady state at 50% where DESeq2, glmGamPoi and CV showed the greatest distance which indicates these metrics were the most influenced by the reduction in cell sample size (Supplementary figure 1). In contrast, other metrics demonstrated substantial variation among replicates under the same sub-sampled sizes, especially for edgeR.”

2. The authors should test their metrics on positive controls with high biological variations (e.g.,  datasets with multiple cell types included vs. a single cell type).

This is a fantastic suggestion! We have extended the metric comparisons to include a series of cell-type mixtures that were created to reflect a range of increasing data complexity. This approach represents a way to include a positive control that has high biological variation. Please see section: *Investigating the impact of known degrees of variability on metric performance.*

See page 9, line 273.

“Additionally, we used different cell-type mixtures with increased data complexity to evaluate the metric comparison [20] (see Methods). This test serves as a positive control to evaluate the performance of the metrics where high biological variation exists. As expected, most of the metrics showed increased or stable overall variability as the complexity of the cell mixtures increased. Metrics which exhibited the most substantial changes in variability were BASiCS, CV, edegR, DESeq2 and glmGamPoi (Supplementary figure 5). “

3. Cells in the UMAP (supplementary figure 8) are separated by clusters and not organized into trajectories.

Yes, the reviewer is correct. We have updated the figure now to show the trajectory as measured from Monocle 3 with a pre-set start on the HSC group. The data has been corrected with age and normalised by scran. These details have been included in the Methods section (see page 22, line 728).

4. For genes that show high or low variability along B cell differentiation, are their stability associated with their expression level in different B cell state?

We have extended our analyses to address this reviewer’s comment by investigating what average gene expression changes occur for genes that show high or low variability along the B cell differentiation process. Please see section titled “Fluctuations in cell-to-cell variability help explain the complex B lymphocyte differentiation process” (see page 11, line 323), where these new analyses are described including Supplementary Table 3 (the high variability genes correspond to those labelled as consistently variable, the low variability genes correspond to the consistently stable genes) and Supplementary Figure 11c. The analysis demonstrates that genes showing consistent changes in variability across the differentiation process do not demonstrate the same significant differences in average expression which further reinforces the importance of gene expression variability for determining transcriptional regulators of differentiation.

These new results are discussed in page 12, line 368:

“Interestingly, these makers showed inconsistent degrees of average gene expression changes that defied the differentiation pattern, especially in HSCs. This result highlights the complementary information that cell-to-cell variability contributes towards identifying regulators of a differentiation process (Supplementary Table 3, Supplementary figure 11c). For example, *Tnfrsf13c* showed high but non-specific average gene expression between the four types of B cells whereas the level of cell-to-cell variability for this same gene reflected high specificity in naïve B cells.”

5. The authors detected slightly decreased variability of gene expression in the aged HSC. Is this in contrast with the previous report that aging is associated with increased transcriptome noise?

Our analysis did reveal a slightly decreased variability in old HSCs with respect to young HSCs based on the distribution of all genes. However, in the external validation where we investigated the overlap in differentially variable genes from TMS with the LT-HSCs and ST-HSCs, we saw more highly variable genes in older versus younger ST-HSCs (and LT-HSCs).

To address this reviewer’s question, we launched a more extensive investigation into the literature surrounding ageing and transcriptional noise. An older study directly tackled the question of whether ageing is associated with increased noise. Warren et al. (2007) titled “Transcriptional instability is not a universal attribute of aging.” investigated six different mRNA transcripts in young mice and found no significant increase in their transcriptional heterogeneity with age in hematopoietic stem cells. We found a more recent study by Kowalczyk et al. (2015) which determined that gene expression variability among HSCs was predominantly associated with the cell cycle where a decrease in the frequency of old HSCs that are in the G1 phase reduced their ability to differentiate. In summary, our results support the premise that the increases that are observed in transcriptional noise during ageing appear to be gene-specific rather than tissue-specific, and this observation complements a recent study by Marti et al (2022): <https://www.biorxiv.org/content/10.1101/2022.06.23.497402v1.full>

We have included some of the above text in the Discussion session. Please see page 18, line 525.

“There are multiple reports of increased cell-to-cell variability with ageing in different cell types and perhaps over time, these have led to the expectation that variability increases in ageing. However, some studies have also reported uncertain or inconsistent levels of cell-to-cell variability changes in ageing even with similar experimental settings. For example, Marti *et al*. applied a quantitative statistical model on the TMS data and found sets of genes which showed a robust decrease of noise with age [44]. One reason could be the biological differences in ageing processes among different species, organs, and conditions, leading to the hypothesis that the increased transcription noise in ageing appears to be gene-specific rather than tissue-specific. In fact, such hypothesises have been validated in many studies especially for HSCs [15, 32, 45]. For example, Kowalczyk *et al.* revealed that gene expression variability among HSCs was predominantly associated with cell cycle and a decrease in differentiation ability during ageing.”

6. How does the target TF (Sfpil) expression level change during aging for HSC and naive B cells? Is the expression changes associated with the variability of this gene?

These are two terrific questions, and we thank the reviewer for raising this point. To address these questions, we identified genes that were differentially expressed between young and old mice in the same dataset for HSC and naive B cells. The TF Sfpil did not have statistically significant changes between young and old mice in either cell type (FDR > 0.01). To better visualise the shifts occurring in variability alongside gene expression level changes, we used a violin plot to represent the gene expression distributions of TF Sfpil, please see Figure 6c (page 16). We have also updated the text in the manuscript to include this newer result regarding the average expression changes of TF (Sfpil) during ageing. Please see page 14, line 445.

“Additionally, we identified a shared TF, Sfpi1, demonstrating consistently increased variability in ageing for both cell types (Figure 6c) that was independent of mean expression difference.”

We also investigated whether the genes targeted by *Sfpi1* demonstrate average expression changes during aging in HSCs and naïve B cells. Interestingly, several DV targets showed statistically significantly changes in gene expression between young and old mice, which emphasises the impact of *Sfpi1* expression variability for controlling downstream gene regulation (Igj and Elane in HSC; Ptprc, Bcl2, Ly9, Cd72 in NB). We have updated the text in the manuscript to incorporate this additional analysis. Please see page 15, line 454.

“Surprisingly, several targets showed significant mean gene expression differences between the young and old groups regulated by fluctuated *Sfpi1* expression. This result emphasises the influence of gene expression variability on controlling regulation which may be overlooked by only detecting mean gene expression changes. (*Igj* and *Elane* in HSC; *Ptprc*, *Bcl2*, *Ly9* and *Cd72* in naïve B cells; adjusted P-value < 0.01; Supplementary figure 12f).”

7. The text fonts in many figures are too small to see (e.g., Supplementary Figures 3, 5, and 7). These should be improved.

We have increased the font size and figure resolution to all main and supplementary figures.

**Reviewer #2**

Zheng et al. have described a benchmarking study in which they have compared 14 metrics for assessing cell-to-cell expression variability in single-cell RNA-sequencing data. After benchmarking all these methods, they have picked the top-performing method (scran) to assess cell-to-cell expression variability during HSC-to-B-cell differentiation and aging in B-cells and HSCs from young vs old mice.

One of the unique properties that can be assessed in scRNA-seq data as opposed to bulk RNA-seq, is the cell-to-cell expression variability. Measuring this could reveal biology that has previously been hidden. However, a systematic comparison of cell-cell expression variability metrics in scRNA-seq data was something that was not conducted before. This has made it difficult so far to interpret studies using different metrics for assessing cell-cell expression variability, and therefore, I believe this study is a valuable addition for the field.

In general, I enjoyed reading the study, and most of the reasoning has been clearly explained. However, there is a few points that could further strengthen the study or enhance readability:

1. The labeling of Figures can be a bit more consistent across figures. For example, Fig. 1 says FACS vs droplet, Fig. 3 10xdroplet vs smartseq2, Fig. 6 3'-end vs full-length, etc. For accessibility for a broader audience, I suggest to use the description: full-length (smartseq2) vs 3'-end (10x droplet).

We thank the reviewer for spotting these inconsistencies and completely agree that it would be clearer to be more consistent. We have adopted the reviewer’s suggestion and relabelled the sequencing platforms as full-length (smartseq2) vs 3'-end (10x droplet) in Figures 1&5 and all supplementary figures.

2. Fig. 1B is a bit difficult for me to interpret. I don't understand what is on the x- and y-axis or how I should interpret the lines etc. I can read the conclusions, but by looking at the figures themselves I couldn't clearly see the conclusions that were drawn (but that might just be me not understanding the actual figures).

We agree with the reviewer that despite our best intentions this figure turned out to be more confusing than clarifying about the data. Ultimately we were trying to communicate the size of the variability due to platform effects (by displaying the distance between HSC on FACs versus Droplet platforms, and similarly for Naïve B cells) and the variability due to the cell type effect (by displaying the distance between HSC versus Naïve B cells on the FACs platform as one distance, and HSCs versus Naïve B cells on the Droplet platform as a second distance). We have replaced this figure with a more straightforward bar plot where these four distances are represented by their own bars (see Figure 2B, page 6, line 181):

Platform variation:

- 3’-end (10X droplet) -- HSC vs Naïve B cells
- full-length (smartseq2) -- HSC vs Naïve B cells

Cell type variation:

- HSC -- HSC full-length (smartseq2) vs HSC 3’-end (10X droplet)
- Naïve B -- Naïve B full-length (smartseq2) vs Naïve B 3’-end (10X droplet)

We hope that this revision helps to communicate our point about the data more clearly. Fundamentally the plot is illustrating the variability due to platform versus the variability due to cell type differences. The distances that are input to the plot are also represented in Supplementary Table 2.

3. Fig. 2B - DM method: what does a negative cell-to-cell variability value implicate here? It's not like you are comparing two conditions, so it's a bit unclear what this means?

This is a very reasonable question, and we thank the reviewer for highlighting this gap in our explanation. We have updated the text to clarify the implication of the negative values that some of the variability metrics have produced. In short, the metrics that are based on a regression model often use residuals as a surrogate for estimating variability and hence, the values can be negative, and the implication is that the variability is lower than expected. Please see page 19, line 582.

“Novel model-based approaches have been introduced to regress unwanted technical variation, to improve the accuracy and detection of ‘true’ biological variation in the gene expression data. More importantly, these metrics apply different regression models to account for the mean-variance relationship and residuals are commonly counted as the cell-to-cell variability measurements. Therefore, genes with negative values represent biological variations that are lower than the expected values and vice versa.”

4. Fig. 3: Based on this Figure, it seems that the performance for Smartseq2 vs 10X

Genomics data differs quite a lot for certain methods. And therefore, I wonder whether a separate ranking should be visualized for these 2 methods instead of averaging the ranking (it's not common that both approaches are used in the same study). In general, the largest sample size seems top performing in each of the tested classes of parameters - however, scran -10x genomics - largest sample size is performing worst. How do you explain this (especially since you're suggestion is to use scran as top performing approach, it would be good to understand when it may deviate)?

This is an excellent point and we agree that providing separate rankings based on sequencing platform would be a way to make our results more pragmatic given that it is usually the case that only one platform is used to generate the scRNA-seq dataset.

We have updated the manuscript to provide the separate rankings (see Supplementary figure 13) rather than combining them together. In the main text, we have kept the average score to give readers an overview of the metrics in general and to keep things simpler. metric performance based on sequencing platform, and in particular we have added further discussion of the potential of using CV to identify stably expressed genes. Please see page 17, line 490 for this additional text.

“Our benchmarking analysis demonstrated that the widely-used metric CV might introduce artificial variations due to the low mean expression resulting from excessive zeros in scRNA-seq data, especially in 10X droplet-based data (Supplementary figure 13). However, CV accurately captured the expected low variability patterns for ribosomal genes and SEGs which suggests its utility for identifying stably expressed genes for those data with relatively low dropout. In fact, a novel method Cepo that identified differential stability was recently developed based on relative CV metric [41].”

Regarding the reviewer’s comment about the concordance between performance and sample size in general, versus the opposite behaviour with scran, we believe this effect is due to the fact that the largest 10k sorted CD19+ B cell dataset (see Methods) was derived from human samples which is expected to have greater donor variability than the Tabula Muris dataset which was done on laboratory-based mice. This effect is the most pronounced for the metric evaluation categories (Figure 5B) mean expression and SEGs, where the performance of the scran metric is worse for the 10X droplet sequencing that were done for the datasets with larger sample size. The larger B cell dataset was included because of the increased sample size (relative to the B cell populations in Tabula Muris Senis) but this also represents a comparison of different species which must be acknowledged. Similarly, the list of SEGs, were curated from various human datasets and for this category it is also important to acknowledge that these datasets consisted of much smaller cell numbers (on average 400 cells per cell type) relative to the larger numbers that were in our comparison. Therefore, the SEGs may not generalize to larger sample sizes as robustly for this reason. We therefore think that these factors, such as donor variability, and sample size, can influence the performance of the metric.

We have updated the main text to better reflect this information so that readers can be more informed from our comparisons. Please see page 7, line 209 for this revised text.

“We also investigated the impact of sample size on the variability metrics using an external set of B cell ultra-large human datasets with more than 10k cells (10X droplet-based) [17]. The 10k B cell data was compared to the B cell (naïve B cell; 50 cells) in TMS droplet-based data (see Methods). Overall, SD, IQR, scran, Seurat and BASiCS showed a reduction in the variability estimated for the 10k B cells compared to the TMS B cell dataset. Surprisingly, CV, FF, DESeq2, DM and edgeR showed increased gene expression variability for the 10k B cells. It is important to note that while the comparisons with this ultra-large dataset suggest that for categories like mean expression and SEGs, the performance of some metrics, including scran, appear to be worse for the datasets with more cells, this trend in performance is likely attributed to the fact that there is a higher degree of donor variability in the human B cell ultra-large datasets than in the laboratory-based mice in the TMS B cell dataset. Moreover, the list of SEGs were derived from human datasets [18] where the sample sizes were relatively smaller (average 400 cells per cell type) than the number of B cells used in this study and this difference may therefore affect the performance observed (Figure 4b).”

5. The labelling of the Figure axes is sometimes absent (but can be extracted from the title of the figure). However, Fig. 4C y-axis for example is unclear.

We have modified the figure to clarify the labelling of figure axes in now Figure 5c (see page 13).

6. Figure 6 gives a nice overview of what has been done in the study, but having it as the last figure is strange for a study overview (then making it Figure 1 would make more sense). It would be very valuable to make this figure a summarizing figure instead, so including advice for the reader which approach to use depending on the dataset characteristics (10X droplet vs FACS smartseq2, dataset size, dropout rate, etc).

We agree with your comments and have moved Figure 6 to Figure 1 to provide an overview of the metric comparison framework.

7. In the following text: 'Overall, there was a slight decrease in variability observed in old HSC compared to young HSC (Wilcoxon test, marginal P-value = 0.04, Supplementary Figure 10a) and no significant effect in naïve B cells (Wilcoxon test, marginal P-value = 0.7, Supplementary Figure 10a).' What is meant by 'marginal p-value'?

The term marginal P-value typically refers to situations when the P-value exceeds the threshold for statistical significance but because it is still numerically very close to this threshold, it is flagged as being marginal as a real effect may potentially be present. The reviewer’s question allowed us to re-evaluate the text that has been flagged and we realise that we have not used the term marginal correctly in this instance. We have removed the term marginal P-value and instead report these P-values as not significant. We thank the reviewer for this question which allowed us to catch this error.

8. 'To increase the power for calculating DV genes, we first pre-filtered the genes based on the proportions of zeros.' Please specify in more detail which cut-off was chosen.

We have updated the Methods section to include the specific cut-off that was used in the differentially variable expression analysis. Please see page 23, line 738.

“To increase the power for calculating DV genes, we first pre-filtered the genes based on the proportions of zeros so that only genes that expressed more than 5% of the total cell population were retained.”

9. Pathway overrepresentation analysis: unclear what background gene set has been used to compare against. Please specify in the methods section.

We have updated the Methods section to specify that the background gene set used in MSigDB was all mouse genes in the Tabular Muris dataset. We agree with the reviewer that this is an important detail to include for reproducibility and transparency of the analyses. Please see page 23, line 762.

“To investigate gene sets at the molecular and functional level, we performed enrichment analysis on GO biological processes pathways by using MSigDB [61] for identifying the biological functions of genes under B cell differentiation pattern and KEGG database by clusterProfiler [62] for revealing biological functions with respect to aged HSCs and naïve B cells, with all genes in TMS data as universe background.”

10. Would be interesting to learn how the DV results compare to the DE results for the same comparison (e.g. aging analysis). Do you identify new sets of genes that weren't detected by one or the other?

We greatly appreciated your ideas on comparing DV genes and DE genes and we have added this analysis for the aging HSC and naive B cells. In fact, we find that at least 20% of DV genes are independent of DE genes, which include the shared TF Sfpi1 we discussed in the main text. Please see page 14, line 422 for this revised text.

“Additionally, we examined whether the DV genes identified in TMS showed differential expression (DE) in terms of mean expression between the young and old groups. While the number of DE genes was substantially higher than the number of DV genes, we found at least 20% unique DV genes with no significant mean differences during ageing in HSCs and naïve B cells (Supplementary figure 12cd and 12e).”

11. How should I interpret the DV results in general? Looking at the results, it seems that many of the genes are associated with subcelltype composition: 'Three cell differential markers, Ccr7, Tnfrsf13c, Cd19, showed the greatest variability changes along the differentiation process'. So is it truely biological variability that increases, or is the accuracy of cell type annotation also disturbing the analyses?

The three cell differential markers highlighted here were identified as part of our consistently variable genes where genes displayed successively increasing variability between HSCs to naïve B cells and the three intermediate cell types in between these two differentiation end-points. It is possible that the increasing variability observed for these differentiation markers reflect the emergence of different subtypes within the cell type category, e.g. new specialized types of naïve B cells, and hence we see more variable expression. It is also possible that the variability reflects more diversity of cell states within naïve B cells. To respond to the reviewer’s question, we conducted a closer inspection of these three differentiation markers and found that despite the successive increases in variability based on cell type during the differentiation process, we saw inconsistent changes in the average expression (see new Supplementary Figure 11C). We think that it is unlikely that cell type annotation accuracy is a factor in this case because of the extensive resources used by the Tabula Muris Senis consortium to apply automated and manual cell annotation labels to this dataset. Instead, it is possible that the variability reflects emergence of new subtype populations within the labelled cell type population as indicated by the reviewer. We have updated the main text to better reflect this interpretation of the data, please see page 12, line 368.

“Interestingly, these makers showed inconsistent degrees of average gene expression changes that defied the differentiation pattern, especially in HSCs. This result highlights the complementary information that cell-to-cell variability contributes towards identifying regulators of a differentiation process (Supplementary Table 3, Supplementary figure 11c). For example, *Tnfrsf13c* showed high but non-specific average gene expression between the four types of B cells whereas the level of cell-to-cell variability for this same gene reflected high specificity in naïve B cells.”

To address the reviewer’s opening question though about DV results and their interpretation, it is worthwhile making the distinction between the analysis of consistently variable genes that these differentiation markers were found from during the differentiation process of HSCs to naïve B cells, versus the DV analysis which contrasted gene expression variability between old and young mice in a specific cell type from the Tabula Muris Senis dataset (see page 14, line 399, and Figure 6). The DV genes are those that had a statistically significant change in their expression variability between young and old mice. We therefore interpret these DV genes as those whose variability change is age-specific.

“To decipher transcriptional heterogeneity changes in ageing, we measured the cell-to-cell variability for HSC and naïve B cells in the young and old groups, respectively (Figure 6a) using scran. We denote these genes as differentially variable (DV) to make the distinction from genes that are differentially expressed between two groups, in this case, young versus old. The DV genes can be interpreted as those genes with a statistically significant change in the variability of gene expression that is age specific.”

On closer inspection we realise that the first time the term DV has been defined was in the Methods section. Hence, we have corrected this oversight and also updated the section that introduces the analysis of the DV genes to ensure readers are clearer on how to interpret these genes.

12. Suppl Table 2: Should the D-statistic be as small as possible when comparing same cell type across scRNA-seq platforms, and as high as possible when comparing different cell types across the same scRNA-seq platform? In other words, are you kind of comparing the influence of technical vs biological variability on the precision of the cell variability metric? Maybe you can aid the reader here by making more clear which comparisons you would like to have an as low as possible vs an as high as possible D-statistic (color-code where green is best/red is worst)?

Yes, the reviewer’s interpretation is correct for expectations surrounding the D-statistic. We have updated Supplementary Figure 2 by highlighting metrics with the good performance in yellow and the poor performance in green. To improve the interpretation of Supplementary Table 2, we include the explanation of the D-statistics in the *Investigating how sequencing methods impact metric performance* section to help reader understand our comparison. Please see page 5, line 168:

“For a well-performed metric, the distance between different sequencing platforms on the same cell type should be substantially lower than the distance between cell types sequenced by the same platform.”

13. Suppl. Fig.1: better to align the last block (largeB droplet) with the top ones, that eases comparison across the different cell types.

We have made the change to reorder the largeB droplet to the top in the new supplementary figure 2.

14. For some figures, e.g. Fig. S4, S5, S6, etc., it is not directly clear just looking at the figure which data has been used as input (especially whether it's smartseq2 or 10x data would be good to know as the performance of some metrics seem to be better for one than the other). Just adding this information in the legend (when it's not already in the figure itself) would be helpful.

We thank you for your suggestions and we have added sequencing platform information into figures.

**Second round of review**

**Reviewer 1**

The authors have commendably addressed all critiques raised by the reviewers, including those put forth by the second reviewer. The introduction of analyses utilizing both positive/negative control datasets, and the validation via external datasets, adds valuable depth to the study. At this juncture, I have no additional comments on the manuscript.
